# Long term environmental variability modulates the epigenetics of maternal traits of kelp crabs in the coast of Chile

**DOI:** 10.1038/s41598-022-23165-1

**Published:** 2022-11-05

**Authors:** Simone Baldanzi, Gonzalo S. Saldías, Cristian A. Vargas, Francesca Porri

**Affiliations:** 1 Laboratorio de Ecofisiologia y Ecologia evolutiva marinas (eCO2lab), Facultad de Ciencia del Mar y de Recursos Naturales, Universidad de Valparaíso, Av. Borgoño 16344, Viña del Mar, Chile; 2 Centro de Observación Marino para Estudios de Riesgos del Ambiente Costero (COSTA-R), Universidad de Valparaíso, Valparaiso, Chile; 3 South African Institute for Aquatic Biodiversity (SAIAB), Private Bag 1015, Makhanda, 6139 South Africa; 4 Instituto Milenio en Socio-Ecología Costera (SECOS), P. Universidad Católica de Chile, Santiago, Chile; 5 Departamento de Física, Facultad de Ciencias, Universidad del Bío-Bío, Concepción, Chile; 6 Laboratorio de Ecosistemas Costeros y Cambio Ambiental Global (ECCALab), Departamento de Sistemas Acuáticos, Facultad de Ciencias Ambientales y Centro de Ciencias Ambientales EULA Chile, Universidad de Concepción, Concepción, Chile; 7 Department of Zoology and Entomology, Rhodes University, Makhanda, 6139 South Africa

**Keywords:** Ecology, Ocean sciences, Epigenetics, Epigenomics, Evolutionary biology, Population genetics, Ecophysiology, Molecular ecology

## Abstract

The methylation of DNA is an environmentally inducible epigenetic mechanism reflecting the short‐term ecological and environmental background of populations. Marine invertebrate populations, which spread along a latitudinal cline, are particularly suitable for profiling DNA methylation, due to the heterogenous environmental conditions experienced. We used the MSAP (Methylation Sensitive Amplified Polymorphism) technique to investigate the natural variation in DNA methylation of different female’s tissues (muscle, gonads, and gills) and early-stage eggs from five populations of the kelp crab *Taliepus dentatus*, distributed along a latitudinal cline in the coast of Chile. We assessed whether, (1) the distribution of DNA methylation profiles can be associated with the temporal variability of long term (18 years) climatologies (sea surface temperature, turbidity and productivity) and (2) the epigenetic diversity of eggs is related to the population-level phenotypic variability of several maternal investment traits (egg volume, egg weight, egg lipids and fecundity). The DNA methylation of eggs correlated positively and negatively with the long term variability in productivity and sea surface temperature, respectively. Furthermore, the diversity of DNA methylation of eggs correlated positively with the population-level phenotypic variability of several maternal investment traits, suggesting a key role of epigenetic mechanisms in generating phenotypic variability at population level for this species. We provide evidence of a strong link between the temporal variability of long term climatologies with the epigenetic profiles of key early ontogenetic traits associated with the maternal investment of kelp crabs. These modulating mechanisms can hence contribute early to phenotypic variability at population levels in response to local and past environmental fluctuation.

## Introduction

Epigenetics study molecular mechanisms that cause changes to gene expression that are not dependent on changes in the DNA sequence^1^. This phenomenon is receiving increasing attention, with both empirical studies and theoretical models, showing that epimutations (i.e., changes in epigenetic state) can induce phenotypic variability in key morphological, physiological, behavioural and life-history traits^2^. Epimutations can appear spontaneously during replication or repair, similar to a mutation in the DNA sequence itself, and persist across generations within a population, or they can be induced to change in response to the environmental variability^3^. Three main mechanisms have been identified as particularly important when assessing epigenetics: the methylation of nucleic acids (DNA and RNA), covalent modifications at histone tails and non-coding RNAs^4^.

DNA methylation is one of the most studied epigenetic mechanisms in plants and animals^4,5^ and involves the addition of a methyl group to a CpG site, a Cytosine base followed by a Guanine base in the DNA sequence. The methylation of DNA is an environmentally inducible mechanism and affects ecological and evolutionary processes at all biological levels, from the individual (phenotypic variation) to the ecosystem level^3^. Changes in DNA methylation can be associated with variation in abiotic factors such as temperature, pH, diet, and chemicals^6^. In invertebrates, DNA methylation is confined to specific gene regions and associated with broad transcriptional patterns, potentially functioning in controlling spurious transcription or fine-tuning of the transcriptome as a whole^7^. Changes in temperature and quality of the diet can modulate DNA methylation in the early stages of marine species, with ontogenetic-dependent variability^8^. In the purple sea urchin, *Strongylocentrotus purpuratus* the parental environments of adults exposed to different temperatures and *p*CO_2_ treatments can be associated with differentially methylated genes in the offspring^9^, indicating a potential link between DNA methylation and phenotypic plasticity in response to environmental changes. DNA methylation is therefore considered as the molecular mechanism that links the environment to the genome of a species or population, thus reflecting the short‐term ecological background of individuals^10^. Upon this perspective, investigating the variation in DNA methylation at the population level, along with classic phylogeographic studies, may help to understand the ecological structuring of populations^10^. It is, therefore, important to understand how epigenetic profiles may vary among natural populations, especially when populations experience environmental heterogeneity such as along latitudinal gradients and/or strong seasonal variability in abiotic conditions^11^.

Currently, population epigenetics have been investigated in plants^5,12^, mammals^13^, birds^14^, fish^15^ and some invertebrates, including marine species^6,16–18^. To date, no study has assessed the variation in DNA methylation in marine invertebrate populations distributed along a latitudinal cline and its potential correlation with maternal investment traits, although there is evidence that geographically separated animal populations can show high epigenetic variability^20^ as well as variation in maternal investment^20^.

The Chilean coast stretches along a broad latitudinal range in the South Pacific, and it is also characterized by a large spatial-environmental heterogeneity in oceanographic conditions, offering the opportunity to investigate variation in DNA methylation profiles of natural marine invertebrates. Associated with major geographical features of the coast, wind-driven upwelling occurs in several sites off northern and central-southern Chile, promoting the upward transport of cold, high nutrient, low oxygen/low pH waters^22–24^. Freshwater runoff from many small rivers in central-southern Chile results in turbid and acidic river plume waters, determining high pCO_2_ conditions in the river-influenced rocky shore environments^24–26^. Therefore, marine biotas inhabiting coastal environments are exposed to a wide range of natural fluctuations of temperature, turbidity, oxygen, and pH/pCO_2_, which may determine tolerance and/or adaptation to local conditions based on their natural range of exposure^23^. Moreover, high nutrients resulting from coastal upwelling regimes can also result in high productivity of both phytoplankton^27^ and increasing abundance and recruitment of dominant kelp species^27,28^, which would also confer tools to cope with stressful conditions, such as extreme events of temperature, hypoxia, and/or low pH conditions e.g.^29,30^.

The subtidal, kelp dominated rocky shores of Chile are inhabited by the kelp crab *Taliepus dentatus* (Milne-Edwards) which shows an extraordinarily extended latitudinal distribution (from 11.9° S to the Chilean Patagonia 51.0° S^20^), experiencing widely different local environmental conditions (e.g., temperature^20^). Previous studies have suggested that biogeographic breaks along the Chilean coast are related to coherence in the spatial structure of surface temperature, food supply (Chl-a) and turbid river runoff over different temporal scales^26^. Although, most studies have explored the biogeographic structure by connecting the temporal dynamic of biophysical processes and larval recruitment (e.g.,^31^), the influence of (potentially) heritable epigenetic changes associated with such biophysical processes and patterns, is understudied. In females of *T. dentatus*, there is evidence of a latitudinal effect on the maternal investment, with increasing fecundity, eggs weight and egg lipid composition at high latitudes during summer^20^. Furthermore, incubation temperatures during gametogenesis negatively affect the size of early egg of *T. dentatus*^32^, hence suggesting a key role of temperature in shaping maternal investment during the early ontogeny of this species. The epigenetic mechanisms related to the complex organism–environment interactions that shape the physiology, phenology and reproductive biology of marine invertebrates on such an environmentally heterogenous Chilean coastline remain unknown for any marine organism.

Here we studied the variation in DNA methylation of different female’s tissues (muscle, gonads, and gills) and early-stage eggs from five different populations of the kelp crab *T. dentatus,* distributed along a latitudinal cline in the coast of Chile. Given that (1) the DNA methylation is an environmentally inducible mechanism and (2) the Chilean coast shows large spatial-environmental heterogeneity in oceanographic conditions, we hypothesised that DNA methylation should be strongly linked to environmental the variability along the cline. We further assessed whether the epigenetic diversity of eggs (within-population variation in DNA methylation profiles) was related to the population-level phenotypic variability of several traits associated with maternal investment (egg volume, egg number, total egg dry mass and egg neutral lipids). We chose the volume and the weight of early deposited eggs because they best represent the environmentally induced maternal investment in offspring^33^. Furthermore, we selected neutral lipids as proxy of maternal provisioning in term of bioenergetic reserve^20,34^. The epigenetic diversity of eggs was expected to positively relate to the phenotypic variability of traits associated with the maternal investment within each population, confirming the key role of DNA methylation on generating phenotypic variability during gametogenesis and development.

## Results

### Environmental variability

**Figure 1:**
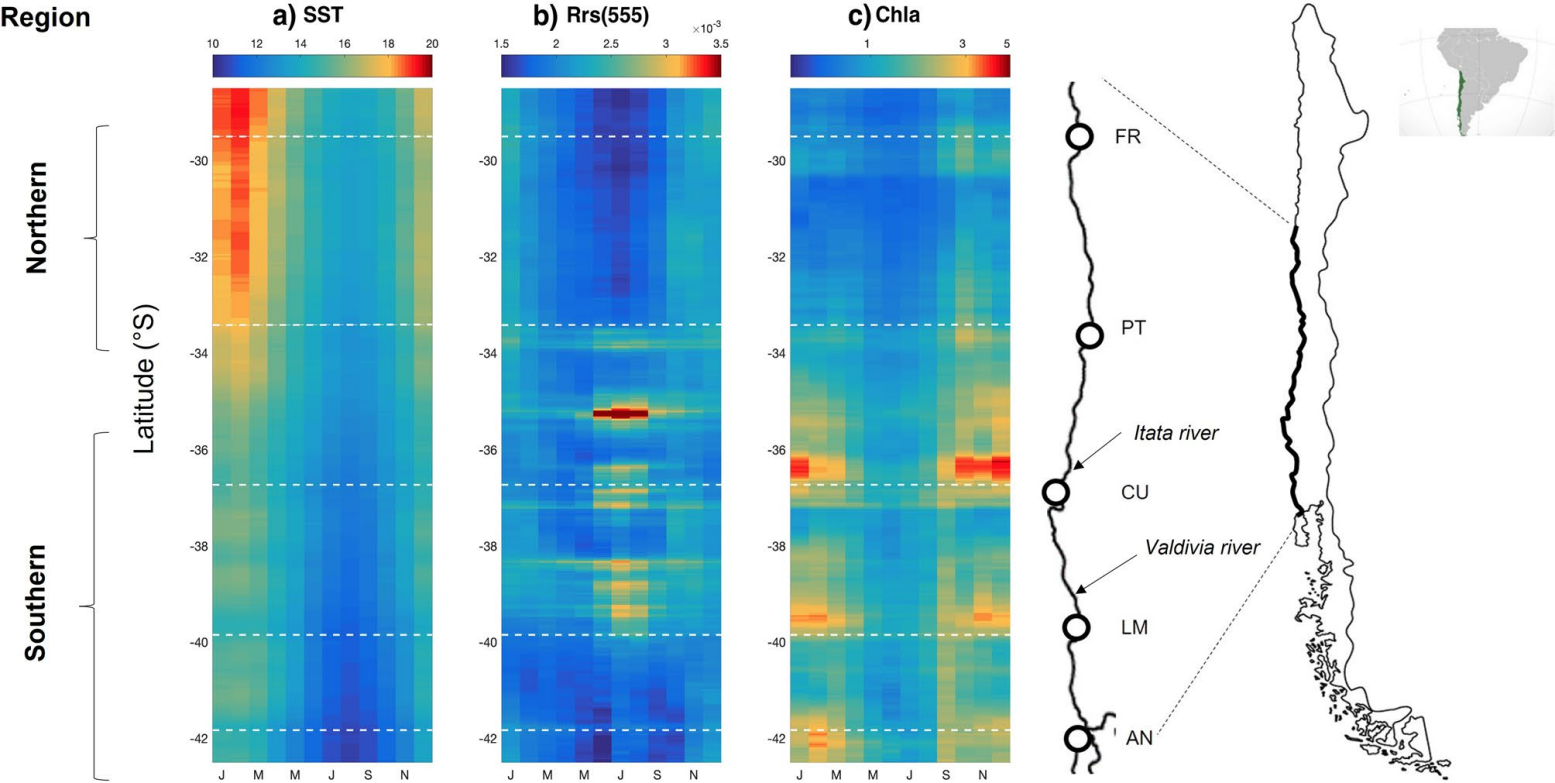
Map of the sampling area (redrawn from^20^) and the long-term (2003–2020) monthly climatologies of the three proxies of biophysical variability. The climatologies (coastal band of 100 km) are represented from January to December along x‐axis, for both (**a**) sea surface temperature (SST, °C), (**b**) turbid river plumes [Rrs(555) (sr‐1)] and (**c**) chlorophyll‐a (mg m‐3). The white horizontal dashed lines denote the location of sampling sites. FR, El Frances; PT, Punta Tralca; CU, Los Cuernos; LM, Los Molinos; AN, Ancud. The map was created using the program Google Earth v7.3 (https://www.google.com/intl/es/earth/versions/) and the software Photoshop v23.4.2 (https://www.adobe.com/cl/products/photoshop.html).

Based on the analysis of satellite imagery we characterized a long term environmental climatology across the *Taliepus dentatus* populations considered in our study. The analysis showed different latitudinal patterns in Sea Surface Temperatures (SST), river-driven turbidity (Rrs(555)) and chlorophyll-a (Chl-a) concentration, with a decreasing trend in SST towards higher latitudes (Fig. 1). The seasonal cycle in SST was more evident in the sampling sites of northern Chile (FR, PT) (Fig. 1a) with the largest variance over the annual cycle (Table 1a). However, when we analysed variability for each season, we observed that the southern sampling locations (LM and AN) experienced the largest variability during summer (Table 1a). The influence of river runoff on environmental variability driven by the turbid river plumes was more evident in those sites belonging to the southern region (CU and LM), mostly associated with the influence of freshwater discharges from Itata and Valdivia rivers (Fig. 1). Finally, the highest environmental variability associated with coastal productivity (Chl-a concentration) was observed in those sites belonging to the southern region, mostly driven by the influence of seasonal coastal upwelling dynamics (CU) and river runoff (LM, and AN) (Fig. 1, Table 1b, c). The analysis for each season in the variability of river-driven turbidity and Chl-a, corroborated the general latitudinal pattern, with the largest variability at CU and LM sites. Nevertheless, the largest variability in this southern region is observed during fall and winter for river-driven turbidity (Table 1b), whereas that the Chl-a concentration showed the highest variability during spring/summer months (Table 1c). Although Chl-a only represents the productivity associated with phytoplankton biomass, fertilization driven by coastal upwelling not only leads to high phytoplankton productivity but also increasing kelp forest growth along the central coast of Chile^28,29^.

**Table 1:** Variance of environmental variables for the 18 years of satellite data at the latitudes of sampling (see white dashed lines in Fig. 1).

(a)	SST				
Sites	Summer	Fall	Winter	Spring	Total
FR	1.29	1.55	0.41	1.43	4.11
PT	1.13	1.34	0.31	1.72	3.46
CU	1.07	0.95	0.24	0.88	1.8
LM	1.89	1.61	0.34	1.32	2.76
AN	1.30	1.28	0.25	1.26	2.58

(b)	Rrs(555)				
Sites	Summer	Fall	Winter	Spring	Total
FR	0.11 × 10^−6^	0.05 × 10^−6^	0.08 × 10^−6^	0.13 × 10^−6^	0.16 × 10^−6^
PT	0.23 × 10^−6^	0.20 × 10^−6^	0.37 × 10^−6^	0.23 × 10^−6^	0.27 × 10^−6^
CU	0.16 × 10^−6^	0.52 × 10^−6^	0.89 × 10^−6^	0.21 × 10^−6^	0.47 × 10^−6^
LM	0.12 × 10^−6^	0.71 × 10^−6^	0.95 × 10^−6^	0.22 × 10^−6^	0.52 × 10^−6^
AN	0.14 × 10^−6^	0.20 × 10^−6^	0.19 × 10^−6^	0.31 × 10^−6^	0.22 × 10^−6^

(c)	Chla				
Sites	Summer	Fall	Winter	Spring	Total
FR	1.01	1.12	6.48	12.07	5.41
PT	2.83	0.32	0.77	12.92	4.51
CU	20.82	1.69	2.15	29.92	14.77
LM	21.24	14.65	5.35	16.63	14.74
AN	10.79	6.87	2.23	19.39	10.61

### Epigenetics and population-level phenotypic traits

**Figure 2:**
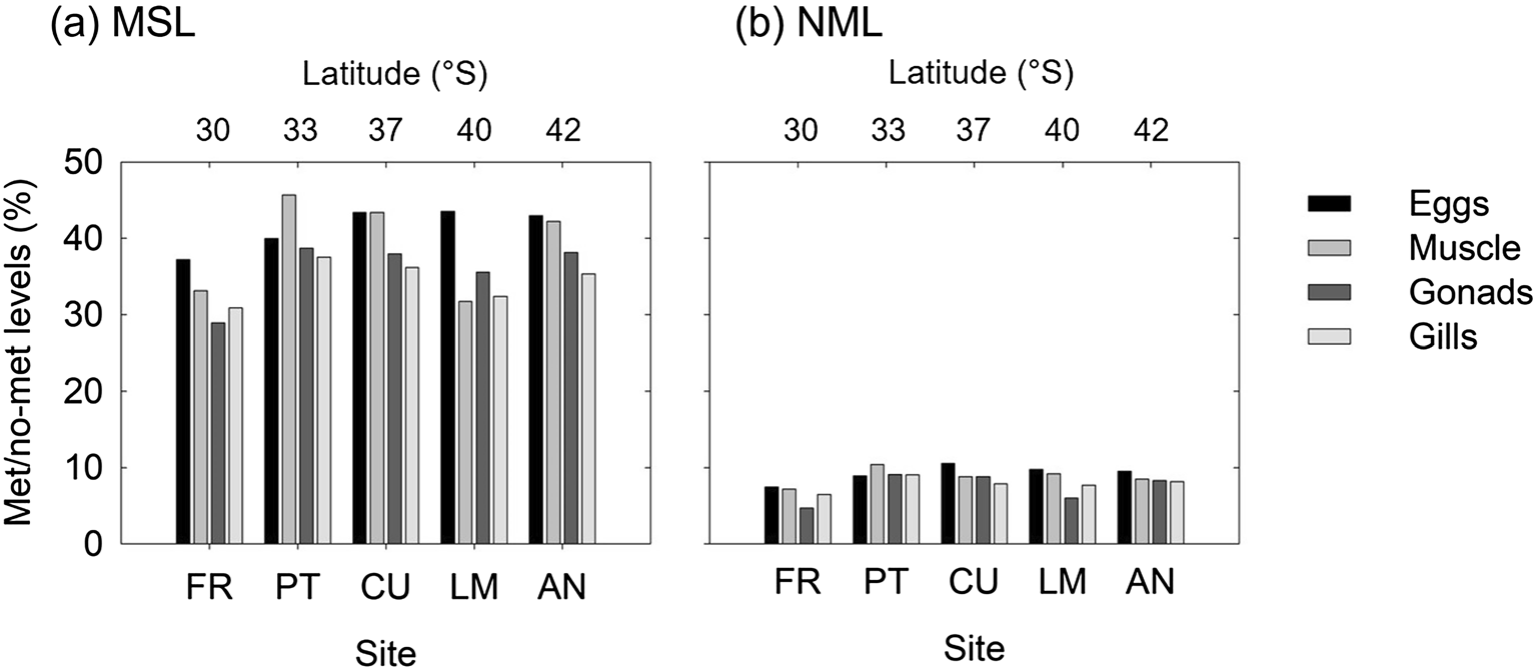
Relative proportions of methylation levels (**a**) and non-methylation levels (**b**) among the five populations for each of the five tissues. FR, El Frances; PT, Punta Tralca; CU, Los Cuernos; LM, Los Molinos; AN, Ancud.

**Figure 3:**
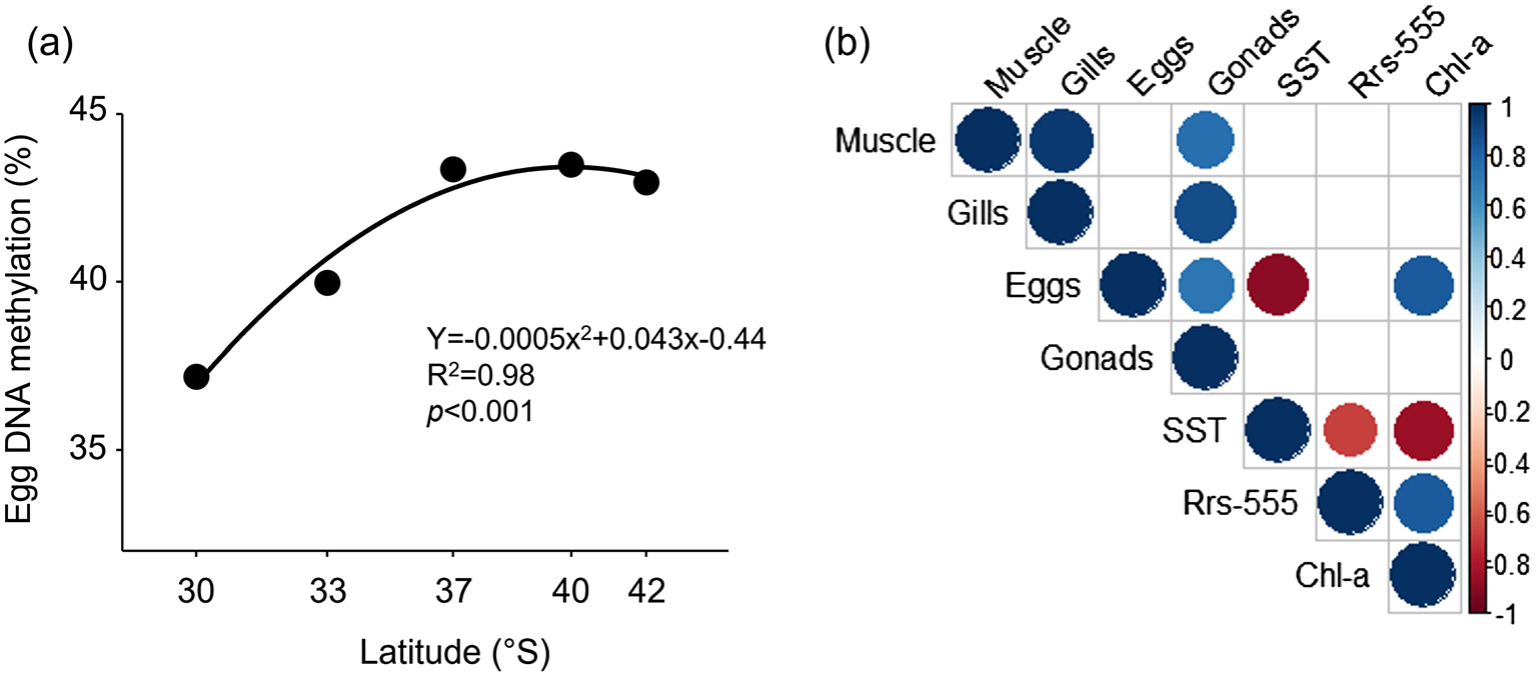
Results of OLS regression between Egg methylation levels and Latitude (**a**) and correlogram (**b**) showing the most correlated variables (Spearman correlations) among the DNA methylation levels (muscle, gonad, gills and eggs) and the climatologies (SST, Rrs(555), and Chl-a). Circles represent significant correlations (*p* < 0.05) between variables, while the absence of a circle (blank cells) represents a non-significant correlation. The size and the colour of the circles in the correlogram represent the correlation coefficient (R) reported in the legend on the right side of the correlogram (− 1 < *p* > 1).

A total of 610 loci were analysed, of which 420 were methylated (MSL) and 190 were non-methylated (NML). The number of polymorphic MSL was 343 (82% of the total MSL), while the number of polymorphic NML was 188 (99% of total NML). The overall methylation levels, calculated from the MSL for the five populations, showed that uninformative sites (HPA−/MSP−) were the most abundant, followed by the methylated sites (sum of HPA−/MSP+ and HPA+/MSP−) and finally by the unmethylated sites (HPA+/MSP+) (Table 2). The levels of methylated loci (MSL) for adult tissues and eggs were always higher than the no methylated ones (NML) at all sampling locations and showed some degree of differentiation among locations (Fig. 2). The DNA methylation of adult tissues (sum of HPA−/MSP+ and HPA+/MSP−) did not follow a latitudinal trend (Fig. 2a) with any of the tissues showing significant relationships with latitude (data not shown). Only the eggs showed a significant, non-linear increase of DNA methylation levels towards higher latitudes (Fig. 3a).

**Table 2:** Overall methylation levels for the five populations.

Site	Latitude	HPA+/MSP+	HPA+/MSP−	HPA−/MSP+	HPA−/MSP−
FR	S 29.95	0.0645	0.1700	0.1522	0.6133
PT	S 33.42	0.09119	0.24087	0.16254	0.50540
CU	S 36.73	0.09362	0.20918	0.19592	0.50128
LM	S 39.85	0.07484	0.16619	0.21000	0.54897
AN	S 41.83	0.08627	0.17952	0.22270	0.51151

HPA+/MSP+, unmethylated; HPA+/MSP−, hemimethylated; HPA−/MSP+, internal cytosine methylation; HPA−/MSP−, uninformative sites; FR, El Frances; PT, Punta Tralca; CU, Los Cuervos; LM, Los Molinos; AN, Ancud.

The DNA methylation of eggs were significantly correlated with the variance of SST (*p* = − 0.89; p = 0.042) and Chl-a (*p* = 0.83; *p* = 0.040), while adult tissues did not show significant correlation with any of the environmental variables (Fig. 3b).

The results of the AMOVAs showed that most of the variance in the MSL was explained within groups (for both tested hypotheses) (Table 3). Different tissues provided different patterns of MSL differentiation among either locations or regions (% variance, Table 3), with the highest among locations/regions variation explained in the eggs, followed by the gonads, muscle and gills. Pairwise ΦST comparisons for the MSL related to the hypothesis 1 confirmed that grouping of MSL among populations differed among female tissue and between tissues and eggs (Table 4).

**Table 3:** Results of AMOVAs for Methylation Sensitive Loci (MSL) for separated tissues and eggs.

Tissue	d.f	SSD	MSD	Variance
**Egg**				
Among locations	4	249.4	62.35	4.78
Within locations	42	1528	36.37	36.37
Total	46	1777	41.15	
Among regions	1	101.4	101.4	2.96
Within regions	45	1676	37.23	37.23
Total	46	1777	38.63	
**Muscle**				
Among locations	4	234.2	58.56	1.65
Within locations	42	1809	43.08	43.08
Total	46	2044	44.43	
Among regions	1	52.24	56.06	0.18
Within regions	45	2525	55.97	56.06
Total	46	2575		
**Gonad**				
Among locations	4	240.3	60.09	2.125
Within locations	41	1665	40.61	40.61
Total	45	1905	42.34	
Among regions	1	46.25	46.25	0.1862
Within regions	44	1859	42.25	42.25
Total	45	1905	42.34	
**Gill**				
Among locations	4	275.7	68.94	1.582
Within locations	43	2312	53.78	53.78
Total	47	2588	42.34	
Among regions	1	56.01	56.01	0.04
Within regions	46	2532	55.05	55.05
Total	47	2588	55.07	

d.f., degree of freedom; SSD, sum of square deviation; MSD, mean of square deviation; Variance, % of variance explained.

**Table 4:** Number of Polymorphic loci, ΦST, pairwise ΦST comparisons among sites for the Methylation Sensitive Loci (MSL) and number of selected MSL after locus-per-locus Chi-squared test (see “Material and methods”) for female tissues and eggs.

Tissue	N° polymorphic loci (% of total)	ΦST	Pairs of sites with significant (*p* < 0.01) pairwise ΦST	N° selected MSL
Egg	256 (61%)	0.0709***	PT ≠ FR, CU, LM, AN	44
Muscle	282 (69%)	0.0369**	AN ≠ FR, PT, LM	103
Gonad	269 (66%)	0.0497***	AN ≠ FR, PT, CU, LM	88
Gill	329 (68%)	0.0286**	AN ≠ FR, PT	61

Asterisks indicate the significance of the ΦST: ***p < 0.0001; **p < 0.01.

FR, El Frances; PT, Punta Tralca; CU, Los Cuervos; LM, Los Molinos; AN, Ancud.

The PCoAs for the hypothesis 1 (single populations as group) confirmed the pattern of AMOVA, with PT providing most of the variation in the eggs (C1 11.7%, C2 7.2%), while AN explained most of the variation for the gonads (C1: 9.8%, C2: 7.1%). Muscle and gonads showed a less differentiated pattern among sites (see Supplementary Fig. S1).

**Figure 4:**
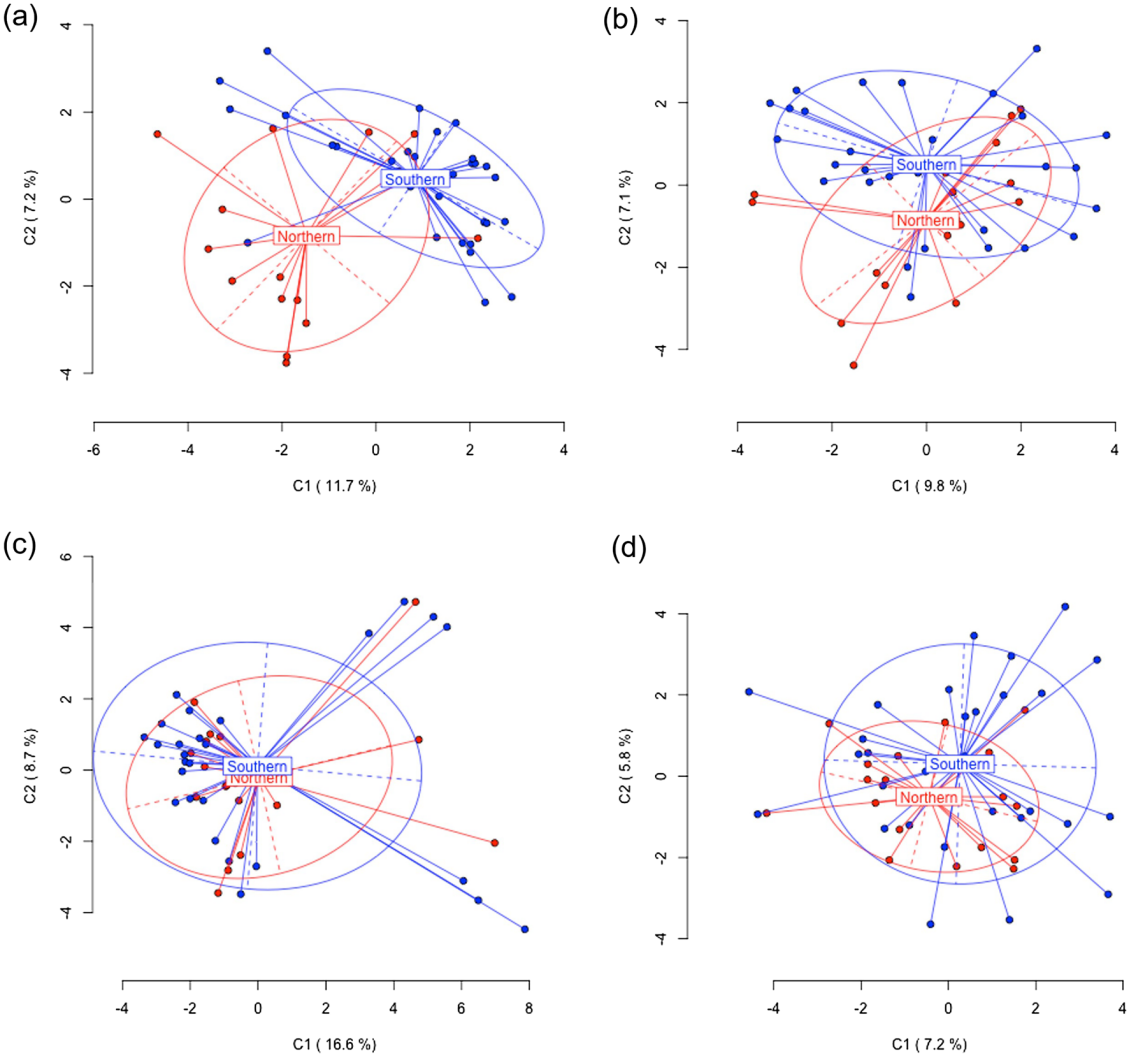
Results from principal coordinate analysis (PCoA) for epigenetic (MSL) of separated tissue (Muscle, Gonad, Gill) and eggs for the two regions (Southern and Northern), after AMOVA results based on hypothesis 2 (see “Material and methods”). The first two coordinates (C1 and C2) are displayed with the indication of the percentage of variance explained in brackets. Scores represent individual samples. Group labels show the centroid for each group (i.e. region). Ellipses represent the average dispersion of those points around the centroid. The long-dashed axis of the ellipse shows the direction of maximum dispersion, and the short-dashed axis shows the direction of minimum dispersion. (**a**) Eggs; (**b**) Gonad; (**c**) Muscle; (**d**) Gill.

The PCoA for the hypothesis 2 (region as group) confirmed the pattern of AMOVA, with Eggs showing most of the separation between regions, followed by Gonads (Fig. 4a and b). Muscle and gills showed a less differentiated pattern between regions (Fig. 4c and d).

Overall (pooling all data), the epigenetic diversity (at MSL) was significantly higher than genetic diversity (NML), Shannon’s index being SI = 0.3558 (SD: 0.1848) and SI = 0.18644 (SD: 0.0772) respectively (Wilcoxon rank sum test with continuity correction: W = 50,142.5, *p* < 0.0001). This pattern of higher epigenetic (MSL) than genetic (NML) diversity was maintained even when the analysis was performed separately for each adult tissue and egg at any location (see supplementary Table S1).

**Figure 5:**
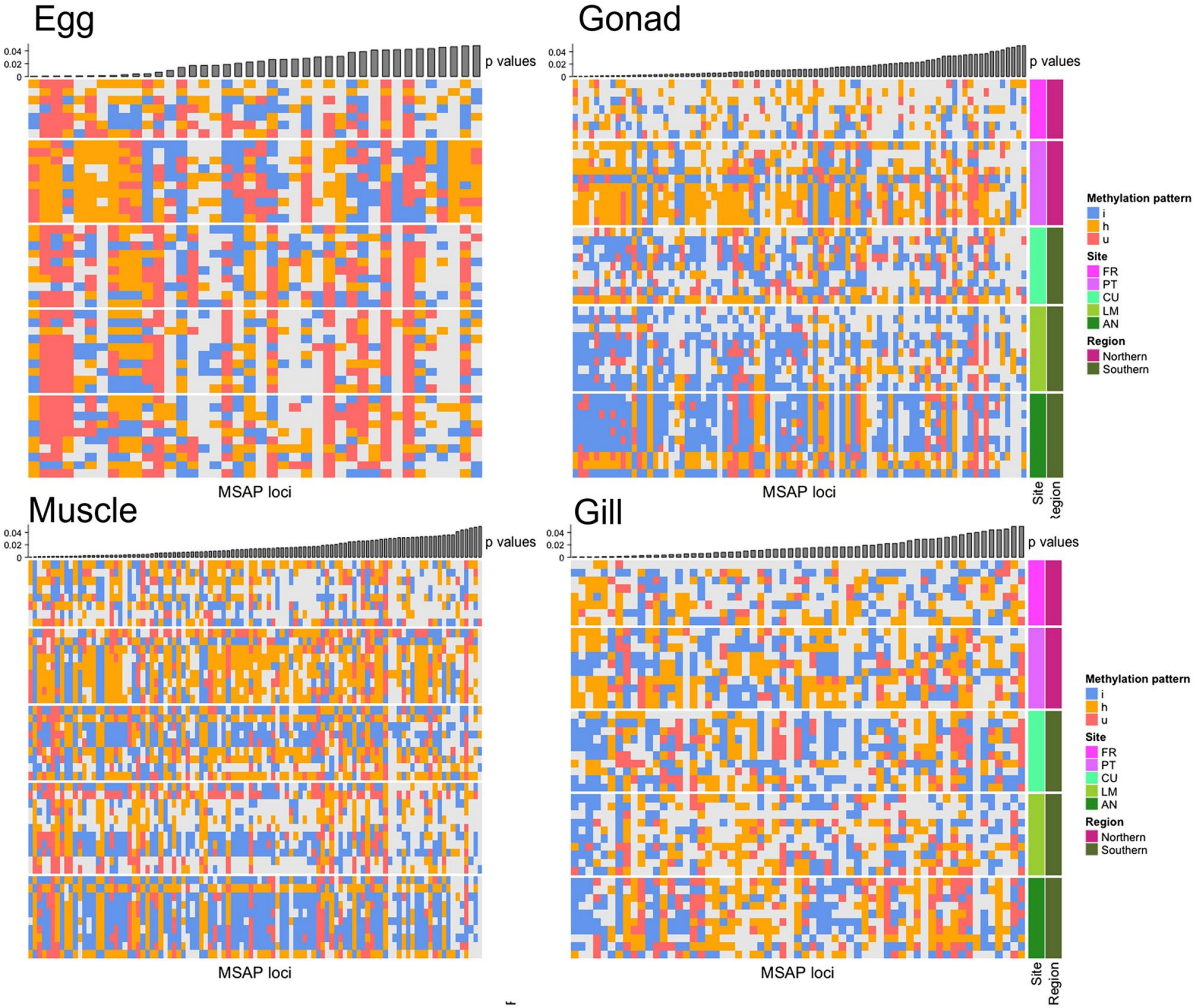
Heatmaps of highly differentially methylated MSL for each adult tissue and eggs. Specimens (rows) and loci (columns) were clustered by the average linkage method. These selected MSL are the most highly differentially methylated loci identified by Chi-squared test after FDR. *p*-values are given on top of each figure. Regions and Site are shown at right side of the heatmap. The colours in the heatmap indicate patterns for the three different methylated states (h: hemimethylated, i: inner cytosine methylation, u: unmethylated) and N/A for uninformative sites associated with each value.

A locus-per-locus chi-squared test yielded different MSL depending on adult tissue and eggs (see Table 4), highlighting different methylation patterns in both regions, with p < 0.001 after a Benjamini and Hochberg^35^ multitest. Figure 5 shows a heatmap with the methylation state for each of the significantly different MSL and samples. As expected, clustering the samples using these MSL resulted in two main clusters, separating Southern from Northern regions, particularly in eggs and gonads. The clustering of the selected MSL showed that all combinations of changes in methylated state were represented, even those implying changes in the type of methylation (h to i or vice versa).

**Figure 6:**
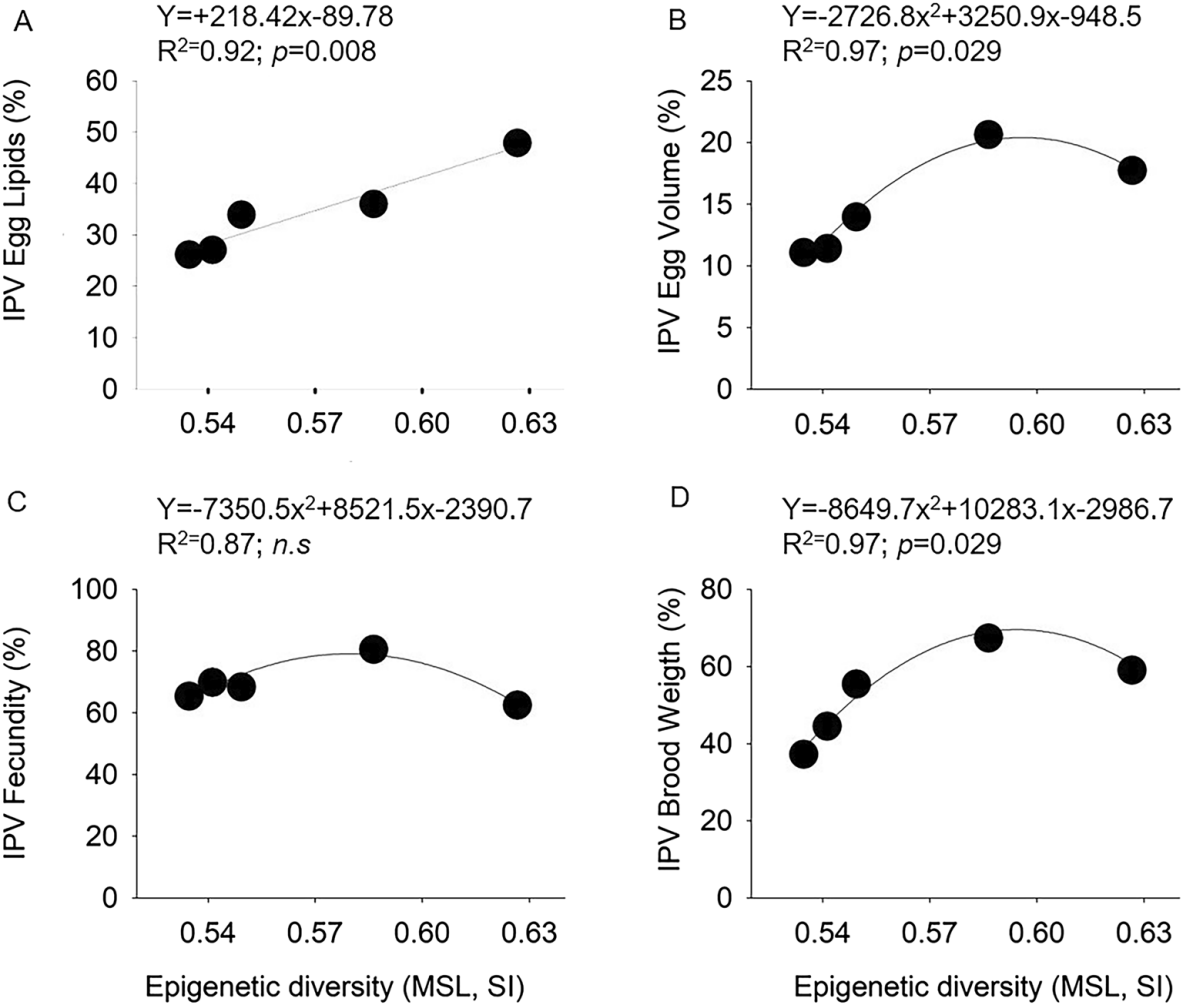
Results of OLS regressions between Interpopulation Phenotypic Variability (IPV) of (**A**) egg lipids and epigenetic diversity, (**B**) egg volume and epigenetic diversity, (**C**) fecundity and epigenetic diversity, (**D**) Brood Dry Weight and epigenetic diversity. The equation of the best fitting model is shown on top of each graph along with the proportion of variance explained (R^2^) and the *p-*values for the significance level (α = 0.05). n.s., no significant; IPV, interpopulation Variability Index; SI, Shannon Index; MSL, Methylation Sensitive Loci.

The IPVs of egg volume, lipids and BDW were significantly related to the egg’s epigenetic diversity (Fig. 6). Particularly, a linear model best explained the increase of the intra population phenotypic variability (IPV) of egg lipids with increasing egg’s epigenetic diversity (Fig. 6A), while a quadratic model best explained the relationship between the IPVs of egg volume and BDW and egg’s epigenetic diversity (Fig. 6B and D). The relationship between IPV of egg number and egg’s epigenetic diversity was not significant (Fig. 6C).

## Discussion

This study explored the variation in DNA methylation of different female adult tissues (muscles, gonads, gills) and eggs within and among populations of a highly abundant decapod crustacean which are separated by hundreds of kilometres along a latitudinal cline. Overall, our results showed that only the proportion of DNA methylation in the eggs increased with latitude. Even though our sample size was limited by the low number of individuals found at the sampling locations, we showed that DNA methylation in females of *T. dentatus* is highly variable, especially among individuals of the same population, supporting the idea that epigenetic mechanisms can produce phenotypic variability. We also showed (1) a tissue-specific pattern in the DNA methylation, with gills exhibiting the highest individual variability in DNA methylation followed by gonads, muscles and eggs, (2) higher variation in methylation-sensitive loci for all tissues within than among population, (3) correlation between egg DNA methylation and long-term environmental variability, and (4) linear and non-linear relationships between the epigenetic diversity of eggs (SI_MSL_) and the intra-population phenotypic variability of several egg-related traits (egg volume, egg lipid content and egg dry weight) associated with the maternal investment of *T. dentatus,* which are known to vary with latitude during summer^21^.

The not-linear relationship of methylation with latitude in the eggs of *T. dentatus* can be explained by either the increased lipid metabolism or food supply at higher latitudes in our sampling area, both associated with decreasing temperatures and increasing productivity^36^. DNA methylation is a well-known process associated with lipid metabolism and synthesis pathway in humans^37^ and, more recently, this association has been determined in fish^38^ and invertebrates^39^. In a laboratory experiment, up to 333 differentially methylated regions between normal and caloric restricted fed individuals of the crustacean *Daphnia magna* were observed, suggesting that epigenetic processes could be involved in the expression of enzymes implicated in lipid synthesis pathways^40^. From a perspective of maternal investment on offspring, more specific studies are required to unravel the close mechanisms that link DNA methylation to lipid metabolism in the early ontogeny of marine invertebrates. Nevertheless, this trend of increasing methylation with latitude also followed the general pattern of productivity along the coast evidenced in our climatologies, which is mostly driven by the influence of wind-driven coastal upwelling events near Punta de Tralca (PT) (Palma et al.^41^) and Los Cuervos (CU) beach in San Vicente Bay^42^ and the influence of nutrients loading from Valdivia River estuary near Los Molinos (LM)^43^ and the Gulf of Ancud (AN)^44^. Although we did not measure the productivity of kelp forests as food supply for *T. dentatus* in upwelling sites, different studies have shown that macroalgae grow faster and attain larger biomass in association with the upwelling sites off Central Chile^28,29^. Moreover, kelp forests are resilient to nutrient loading from freshwater discharges and may experience enhanced productivity when stimulated by nitrogen runoff^37^. Therefore, food supply could be associated with this trend of potential increasing eggs lipid metabolism along the study area.

Tissues displayed different levels of DNA methylation when all individuals within the same population were pooled, suggesting that individual (within-population) variability plays an important role in shaping the population epigenetic profiles of all tissues (including eggs) of *T. dentatus*. Using the MSAP technique, tissue-specific differences in DNA methylation have been recorded in scallops^45^, although with smaller variation within the tissue (20.5–21.4%) compared to the present study (roughly from 30 to 45%). The gill was presented with the highest individual variability, followed by gonads and muscles. This tissue-specific epigenetic difference could be due to the tissue turnover and to the differential spatial–temporal scales at which tissues are exposed and respond to environmental fluctuations during an individual lifetime^46^. Feinberg and Irizarry^47^, using an inherited stochastic variation model, suggested that epigenetic variation can increase in fluctuating environments. Our observations of higher individual variability in tissues that are potentially linked to increased fluctuation in environmental variables agree with the prediction of the model. Such high variation in DNA methylation within populations was further confirmed by the low variation among populations (with a slight exception for the eggs, see below), suggesting that local variability could be more important than large scale variation in environmental conditions or available resources which directly influence the DNA methylation of tissues.

The linear and the non-linear positive relationships between the epigenetic diversity of eggs, expressed as the Shannon Index of MSL, and the intra-population phenotypic variability of several egg-related traits, is noteworthy and suggests a link between DNA methylation and within-population reproductive plasticity. Epigenetic mechanisms have been proposed as core to plasticity, allowing environmental exposure to shape future gene expression^3^. We have shown that increasing the diversity of DNA methylation triggers an increase in the proportion of egg neutral lipids (energy storage) by females of kelp crabs, suggesting that the environment, by acting on the diversity DNA methylation, may be likely responsible for activating/deactivating a portion of genes associated with lipid metabolic pathways. Whether the parental environment or the environmental conditions directly experienced by early eggs are responsible for triggering such epigenetic-mediated increase in lipid storage, was outside the scope of this study. Nonetheless, given that stored lipids in early eggs are associated with the maternal investment^34^, the parental environment likely played an important role in shaping offspring reserves through DNA methylation, strongly supporting the idea that epigenetic mechanisms are associated with heritable plasticity in invertebrates^6^. The non-linear positive relationship between DNA methylation diversity and plasticity in egg volume and brood dry weight also suggests, and possibly in a more evident way, that maternal effects, plasticity, and epigenetics are closely linked.

In conclusion, we found strong links between egg epigenetic profiles and population phenotypic variability in egg related traits. This relationship suggests that phenotypic variation among populations of this species could be shaped by epigenetic diversity. Variability in DNA profiles of eggs showed a correlation with the environmental variability of important environmental variables (Chlorophyll and temperature) that characterise the large-scale oceanographic heterogeneity of the Chilean coast. Our climatology analysis, based on the environmental data of the past 18 years, reflected the role of the environment in shaping current days epigenetic processes during oogenesis and egg brooding in *T.dentatus.* These modulating mechanisms can hence contribute early to phenotypic variability at the population level in response to local and past environmental fluctuation, suggesting that maternal effects may be an important source of environmentally inducible phenotypic variability.

## Material and methods

### Study area and animal collection

Animals were collected during austral summer (December 2015–February 2016) from five geographically separated populations (spanning approximately 1500 km; Fig. 1) during the same surveys and sampling season described in Baldanzi et al.^21^. Populations were chosen to best represent the large latitudinal gradient, known existence of formerly described kelp forests and similarity of coastal upwelling conditions (weak upwelling; see^48^). From North to South: El Frances (FR, S 29.95); Punta de Tralca (PT, S 33.41); Los Cuervos (CU, S 36.73); Los Molinos (LM, S 39.85); Ancud (AN, S 41.83). The use of individuals from the same populations and same survey as described in Baldanzi et al.^21^ allowed for a direct comparison of DNA methylation of females and eggs from this study with the variability in phenotypic traits associated with female investment in eggs of *T. dentatus* (egg lipid composition, egg volume, egg number and brood dry weight) retrieved from Baldanzi et al.^21^.

Ten similarly sized adult females (carapace width range: 50–60 mm) carrying early-stage eggs (see^21^) were collected by scuba divers at each location (average depth of 10 m), immediately frozen and transported to the laboratory where they were stored at − 20 °C until processed. Animals were wet weighed using a precision balance to the nearest 0.0001 g and sized (carapace width and carapace length) before dissection. Three different tissues (muscle from the chelae, gonads and gills) were removed, washed with millyQ water and stored in 96% ethanol until further processing. Additionally, a subsample of about 0.05 g of eggs wet weight was randomly collected and stored in 96% ethanol.

### Environmental data

18 years (2003–2016) of satellite‐derived sea surface temperature (SST), remote sensing reflectance at 555 nm (Rrs(555)), and chlorophyll‐a (Chl-a) from the MODIS sensor on NASA's Aqua satellite (http://oceancolor.gsfc.nasa.gov/) were used to characterize the dominant oceanographic conditions along the latitudinal gradient. All composites correspond to monthly averages (level 3) with a spatial resolution of 4 × 4 km. We used SST and Chl-a as proxies of oceanographic forcing (e.g. coastal upwelling) and phytoplankton productivity, and Rrs(555) as a proxy of turbid river plumes (e.g.^26^) to assess multiscale regimes of biophysical variability along the study area. All satellite data were averaged in the cross‐shore direction (100 km next to the coast) to compute the seasonal cycles as a function of latitude. We also computed the variance as a measure of the temporal variability at each sampling site (Table 1).

### DNA isolation and MSAP genotyping

The protocol used for isolation and genotyping was adapted from Watson et al.^18^. Specimens were dry bathed at 37 °C for 2 h prior to start with the digestion reactions, to avoid the presence of ethanol. As previously outlined in^18^, total DNA was extracted from each tissue (muscle, gills, and gonads) and from the eggs, each purified separately using standard DNA extraction kit (Qiagen DNeasy Blood and Tissue Kit, Qiagen, Hilden, Germany). DNA quality was verified by electrophoresis on 1% agarose gels and DNA quantification was done using a Nano Drop 2000 spectrophotometer (Thermo Scientific, Wilmington, Delaware, USA) with a sample dilution of 100 ng/μl.

The Methylation Sensitive Amplified Polymorphism (MSAP) technique^49^ was performed to detect variation in the DNA methylation among and within the five populations (see^17^for details on MSAP). A first digestion reaction was performed in a total volume of 50 μl containing approximately 50 ng/μl of DNA and 1 U of *EcoRI* (incubation: 37 °C for 1 h; inactivation: 65 °C for 20 min). The mixture was subsequently split in two aliquots to perform a second digestion. Each aliquot contained 25 μl of product and 1U of either *HpaII* or *MspI* (incubation: 37 °C for 1 h; inactivation: 65 °C for 20 min for *HpaII* and 80 °C for 20 min for *MspI*)*.* After digestion, ligation reactions were performed in a total volume of 100 μl containing 10 μl *EcoRI* linker, 10 μl *HpaII/MspI* linkers, 20 U T4 DNA ligase (KapaBiosystems, Boston, Massachusetts) and 10 μl ligase 10 × buffer (KapaBiosystems). Overnight incubation followed at 4 °C. As specified in^17^ a specific pre-selective PCR reaction was performed, followed by an amplification reaction. The pre-selective PCR was done using 5 μl of all ligation products (both *MspI* and *HpaII*) in 50 μl volumes, containing 0.36 mM MgCl2, 0.2 μM of Hpa + A and Eco + A primers, 1 × Buffer (KapaBiosystems) 0.8 mM dNTPs, 1 U Taq (KapaBiosystems). Cycling conditions were the following: 1 cycle of 94 °C for 5 min; 30 cycles of 94 °C for 30 s, 56 °C for 1 min, 72 °C for 1 min; 1 final cycle of 72 °C for 1 min. DNA quality was verified by electrophoresis on 1% agarose gels.

Amplification reactions were performed with 5 μl of diluted pre-selective PCR products (dilution rate of 1:20). The total reaction volume was 20 μl, containing 0.36 mM MgCl_2_, 0.25 μM EcoRI-AG and EcoRI-AC selective primers, 0.25 μM fluorescent primers (*HpaII*-ATT, *HpaII*-ATG, *HpaII*-AAC, Hpa2-AAG), 1 × Buffer (KapaBiosystems), 0.8 mM dNTPs, 1 U Taq DNA Polymerase (KapaBiosystems). Cycling conditions were the following: 1 cycle of 94 °C for 5 min; 12 cycles of 94 °C for 30 s, 65 °C to 56.6 °C for 1 min (decreasing by 0.7 °C per cycle), 72 °C for 1 min; 23 cycles of 94 °C for 30 s, 56 °C for 1 min, 72 °C for 1 min. 1 final cycle of 72 °C for 1 min. *HpaII* digested fragments were amplified using VIC-labelled primers, while *MspI* digested fragments were amplified using PET-labelled primers. Products of the selective amplifications (after combining the *HpaII*- and *MspI*-labelled products) were multiplexed for analysis on an ABI-Hitachi 3500 Genetic Analyser (Applied Biosystems, Austin, Texas). HiDi formamide (Applied Biosystems) and an internal lane size standard (600LIZ; Applied Biosystems) were added to each multiplexed sample prior to analysis and the sizing of fragments.

Following Watson et al.^18^ and Baldanzi et al.^19^, the fragment analysis and scoring were performed using GeneMapper v5 software (Applied Biosystems). To avoid low levels of reproducibility, we excluded those DNA fragments less than 50 bp in length, longer than 500 bp or less than 120–140 RFU (Relative Fluorescent Units). A binary matrix of the presence or absence of fragments in each of the *Eco*RI + *Hpa*II, and *Eco*RI + *Msp*I digests was produced for analysis (the whole genotype matrix is available as Supplementary Data S1).

The analysis of the MSAP profiles summarised in the binary matrix was performed using the *msap* v.1.1.9 package for the R environment^50^. A reproducible example of the script used for the analysis is provided as Supplementary Methods S1. The MSAP fragments were scored as follows: the presence of both EcoRI–*HpaII* and EcoRI–*MspI* products (pattern HPA+/MSP) was defined a non-methylated state, the presence of only one of the EcoRI–*HpaII* (HPA+/MSP−) or EcoRI–*MspI* (HPA−/MSP+) products represent methylated states (hemimethylated or internal C methylation) and the absence of both EcoRI–*HpaII* and EcoRI–*MspI* products (HPA−/MSP−) in a particular individual was considered as an uninformative state, as this can be due to either lack of target or hyper-methylation^50^. All fragments were then classified as either Methylation-Susceptible Loci (MSL) or to Non-Methylated Loci (NML), depending on whether the observed proportion of methylated states across all samples exceeded a 5% error rate-based threshold. MSL are used to assess epigenetic variation, whereas NML are used to assess genetic variation (see^21^) as their banding pattern is assumed to depend exclusively on changes of the sequence at the restriction target, like AFLPs^51^.

Furthermore, locus-per-locus chi-squared tests were performed on the MSL for each adult tissue (muscle, gonad and gill) and eggs to check for which loci the different methylation patterns (h, i, u) were not randomly distributed between the two groups of populations (Regions). As the number of samples is quite low for robust p-values from chi-squared tests, we selected as significantly differentiated only those MSL with p < 0.001 after a Benjamini and Hochberg multitest correction^36^. Estimates of relationships among those highly differentiated loci between sites were computed by Gower’s Coefficient of Similarity. The resulting matrix was clustered using the complete linkage method and visualised as a heatmap matrix using the R package Complex Heatmap^51^.

### Population-level phenotypic analyses

Population-level data of phenotypic traits associated with the maternal investment in eggs (egg volume, egg number, brood dry weight, and egg neutral lipids) were retrieved from Baldanzi et al.^21^ and reanalysed to calculate phenotypic variability at the population level. Briefly, the egg volume (mm^3^) was calculated by averaging the volume of three subsamples of 0.05 g (wet weight) of eggs per female (n = 10) per population. The egg number (used as a proxy of fecundity) was the total number of early-stage eggs estimated per female (n = 10) per population. The total number of eggs was corrected by dividing it by the dry weight of the females to account for female size and providing a standardised measure of fecundity. The total Brood Dry Weight (BDW) is given as the average dry weight of the entire egg mass (g) per female (n = 10) per population. The egg neutral lipids were expressed in non-dimensional units, calculated as the concentration in grams of neutral lipids (the sum of triacylglycerols, cholesterol and monoacylglycerols) corrected by egg dry weight per female (n = 10) per population (see^21^).

An index of intra population phenotypic variability (phenotypic plasticity index sensu^52^, IPV) was calculated for each maternal investment trait as the percentage of change in high and low values, relative to a given trait:

$${\text{IPV}} = \, [(Xmax - Xmin)/Xmax] \times {1}00$$

where *Xmax* is the highest value for a given trait and population and *Xmin* is the lowest value for a given trait and population. This index has been used for comparison of plasticity where the focus is on the responses of species to variable environments and allows for comparisons among traits expressed in different units^52^.

### Statistical analysis

To investigate whether the epigenetics of natural populations of *T. dentatus* vary with latitude, the total amount of methylation (sum of HPA+/MSP− and HPA−/MSP+) for all populations and tissues (included eggs) were plotted against latitude and fitted using Ordinary Least Square (OLS) regressions. By doing so, emphasis was placed on the geographic trends rather than on the individual sites chosen for the study.

To assess whether the variability of the selected environmental variables (variance of SST, Rrs (555), and Chl-a) was a good predictor of DNA methylation of female tissues and eggs, we performed multiple correlation analysis on the average values of each variable.

Multilocus epigenetic differentiation was assessed by the principal coordinates analysis (PCoA) method with subsequent testing with two Analyses of Molecular Variance (AMOVA) using 10,000 permutations^53^. The two AMOVAs were performed considering two different hypotheses: (1) a priori hypothesis of no differentiation in DNA methylation among individual populations and (2) a posteriori hypothesis of no differentiation between two regions, based on the results of environmental variance. The two regions were: the Northern region, a group including FR and PT, and the Southern region a group including CU, LM and AN (see Fig. 1). Shannon’s diversity Index (SI) was calculated for each locus to estimate the amount of epigenetic and genetic diversity within each population using the formula SI = − ΣPi log_e_(Pi), where Pi is the frequency of the presence or absence of the band (i = 1, 2). The mean SI per population is given by an average of the index values over individual loci. A Wilcoxon Rank Sum test was used to determine statistical significance for the comparison between genetic and epigenetic diversities for the five populations. Data were analysed using the *msap* v.1.1.9 package for the R environment (R Development Core Team 2014)^51^.

To investigate whether intrapopulation phenotypic variability of traits associated to the maternal investment in eggs (IPVs of egg volume, egg number, BDW and egg neutral lipids) relate to the epigenetic diversity (SI of MSL) of all populations of *T. dentatus,* the IPVs were plotted against the SI of MSL. Data were fitted for both linear and quadratic regressions using Ordinary Least Square (OLS) regressions.

## Supplementary Information

Supplementary Information 1.

Supplementary Figure S1.

Supplementary Information 3.

Supplementary Table S1.

## Data Availability

All data generated or analysed during this study are included in this published article (and its Supplementary Information files).
