# Supplementary material for: Long term environmental variability modulates the epigenetics of maternal traits of kelp crabs in the coast of Chile
Source: Sci Rep. 2022 Nov 5;12:18806. doi: 10.1038/s41598-022-23165-1 (PMC9637151; doi:10.1038/s41598-022-23165-1)
Supplement: Supplementary file 2 — Supplementary Figure S1. [file 41598_2022_23165_MOESM2_ESM.pdf]

Long term environmental variability modulates the epigenetics of maternal traits of kelp crabs in the coast of Chile.

Simone Baldanzi, Gonzalo S. Saldías, Cristian A. Vargas, Francesca Porri

Supplementary Figure 1

Results from principal coordinate analysis (PCoA) for epigenetic (MSL) of separated tissue(Muscle, Gonad, Gill) and eggs for the five locations.

The first two coordinates (C1 and C2) are displayed with the indication of the percentage of variance explained in brackets. Scores represent individual samples. Group labels show the centroid for each group (i.e. locations). Ellipses represent the average dispersion of those points around the centroid. The long-dashed axis of the ellipse shows the direction of maximum dispersion and the short-dashed axis shows the direction of minimum dispersion. FR: El Frances; PT: Punta Tralca; CU: Los Cuernos; LM: Los Molinos; AN: Ancud.

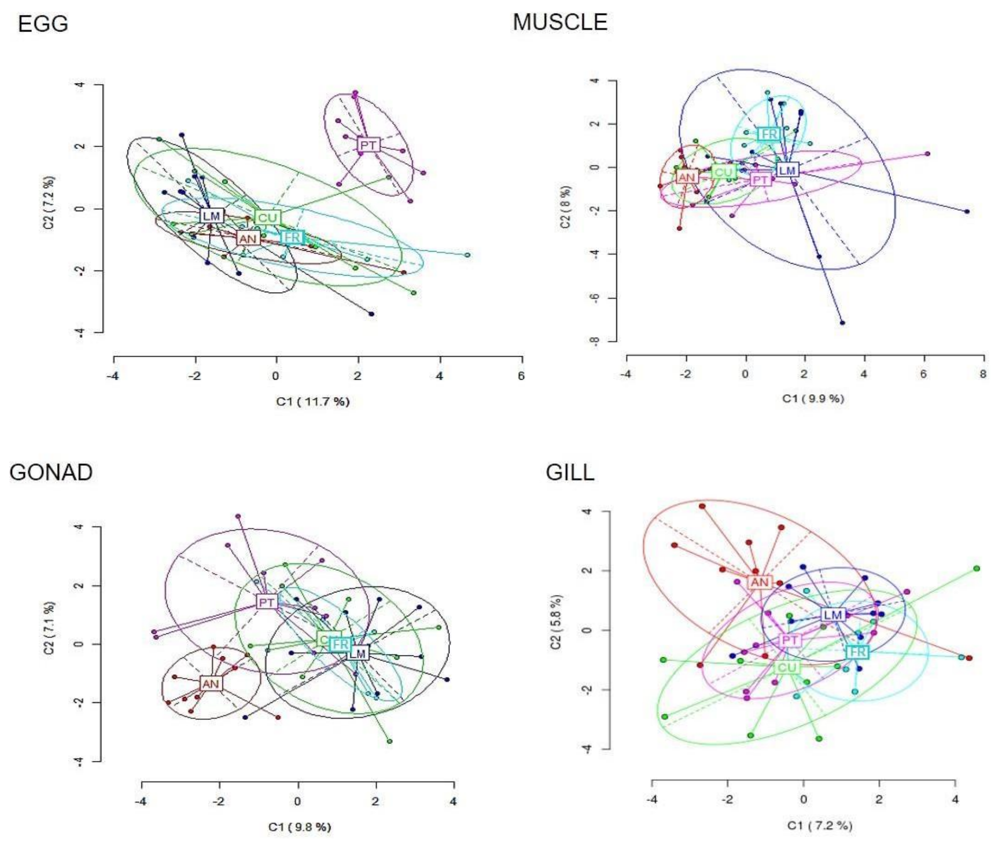
EGG
PT
LM
CU
AN
FR
C2 (7.2 %)
C1 (11.7 %)
MUSCLE
FR
AN
CU
PT
LM
C2 (8 %)
C1 (9.9 %)
GONAD
PT
CU
FR
LM
AN
C2 (7.1 %)
C1 (9.8 %)
GILL
AN
LM
PT
CU
FR
C2 (5.8 %)
C1 (7.2 %)
