# Supplementary material for: Long term environmental variability modulates the epigenetics of maternal traits of kelp crabs in the coast of Chile
Source: Sci Rep. 2022 Nov 5;12:18806. doi: 10.1038/s41598-022-23165-1 (PMC9637151; doi:10.1038/s41598-022-23165-1)
Supplement: Supplementary file 3 — Supplementary Information 3. [file 41598_2022_23165_MOESM3_ESM.pdf]

Long term environmental variability modulates the epigenetics of maternal traits of kelp crabs in the coast of Chile.

Reproducible report

Simone Baldanzi, Gonzalo S. Saldías, Cristian A. Vargas, Francesca Porri

This document reproduces and supports the statistical analysis of MSAP data described in our paper entitled: Long term environmental variability shapes the epigenetic profiles in adults and eggs of the kelp crab Taliepus dentatus along a latitudinal cline in the coast of Chile.

Description of the data files

Provide a link to the required files

Preparation steps

Firts of all, to sucessfully reproduce this document you should use R version 3.6.0

```
#If devtools is not installed, you'll need to install it
if (!"devtools" %in% rownames(installed.packages())) {
  install.packages("devtools")
}

#Install specific version of msap from github
if (!"msap" %in% rownames(installed.packages())) {
  devtools::install_github("anpefi/msap/pkg/msap@v1.1.9")
}

#Load msap
library(msap)
# (Install and)Load other required packages
if (!"ComplexHeatmap" %in% rownames(installed.packages())) {
    if (!requireNamespace("BiocManager", quietly = TRUE))
    install.packages("BiocManager")

    BiocManager::install("ComplexHeatmap")
}
library(ComplexHeatmap)
```

```
## Loading required package: grid

## ========================================
## ComplexHeatmap version 2.2.0
## Bioconductor page: http://bioconductor.org/packages/ComplexHeatmap/
## Github page: https://github.com/jokergoo/ComplexHeatmap
## Documentation: http://jokergoo.github.io/ComplexHeatmap-reference
##
## If you use it in published research, please cite:
## Gu, Z. Complex heatmaps reveal patterns and correlations in
multidimensional
##   genomic data. Bioinformatics 2016.
## ========================================

if (!"ggplot2" %in% rownames(installed.packages())) {
  install.packages("ggplot2")
}
library(ggplot2)
if (!"here" %in% rownames(installed.packages())) {
  install.packages("here")
}
library(here)

## here() starts at /Users/andres/Dropbox/tasks/Taliepus

suppressPackageStartupMessages(library(tidyverse))
```

Geographical (latitude) distance between localities

here, we obtained the distance matrix (in latitude degrees) between the different localities

```
locality <- c("FR","PT","CU","LM","AN")
Latitude <- c(-29.95,-33.41,-36.73,-39.85, -41.83)
names(Latitude) <- locality
dist_latitude <- dist(Latitude)
dist_latitude

##       FR    PT    CU    LM
## PT  3.46
## CU  6.78  3.32
## LM  9.90  6.44  3.12
## AN 11.88  8.42  5.10  1.98
```

msap analysis by tissue

Here, we present the raw analyses of msap for the four tissues analysed ## Eggs

```
res_EG <- msap(here("raw_data","Genotypes Table_Eggs.csv"),
name="EG",do.pairwisePhiST = T,do.mantel = T)

##
## msap  1.1.9  - Statistical analysis for Methylation-Sensitive
Amplification Polimorphism data
##
## Reading  /Users/andres/Dropbox/tasks/Taliepus/raw_data/Genotypes
Table_Eggs.csv ..... Ok!
## Number of loci:  610
## Number of samples/individuals:  47
## Number of groups/populations:  5
## Number of primer combinations:  1
## Loci per primer combinations  610
## Error rates per primer combination:  0.05
## Primer:  1
## --Number of Methylation-Susceptible Loci (MSL):  418
## --Number of No Methylated Loci (NML):  192
##
## All combinations:
## Number of Methylation-Susceptible Loci (MSL):  418
## Number of No Methylated Loci (NML):  192
##
## Number of polymorphic MSL:  256  ( 61 % of total MSL)
## Number of polymorphic NML:  144  ( 75 % of total NML)
##
## - Saving transformed matrix for MSL in file:  EG-MSL-transformed.csv
## - Saving transformed matrix for NML in file:  EG-NML-transformed.csv
##
## Shannon's Diversity Index
## MSL: I =  0.4035468   (SD:  0.1824195 )
## NML: I =  0.1984176   (SD:  0.07253319 )
## Wilcoxon rank sum test with continuity correction :  W  =  30310  ( P
< 0.0001 )
##
##
## *****************************
## Analysis of MSL
## Report of methylation levels
##                                                           AN     CU
FR
## HPA+/MSP+ (Unmethylated)                             0.09498 0.1053
0.07416
## HPA+/MSP- (Hemimethylated)                           0.25215 0.2684
0.19446
## HPA-/MSP+ (Internal cytosine methylation)            0.17751 0.1651
0.17738
## HPA-/MSP- (Full methylation or absence of target) 0.47536 0.4612
0.55400
##                                                           LM     PT
```

```
## HPA+/MSP+ (Unmethylated)                          0.09737 0.0890
## HPA+/MSP- (Hemimethylated)                        0.21244 0.2474
## HPA-/MSP+ (Internal cytosine methylation)         0.22249 0.1522
## HPA-/MSP- (Full methylation or absence of target) 0.46770 0.5115
## 
## 
## - Saving clustering tree figure for MSL in file:  EG-MSL-NJ.png .....

## Ok!

## Warning in cmdscale(DM, k = length(inds) - 1, eig = T): only 44 of the
first 46
## eigenvalues are > 0

## - Saving PCoA figure for  MSL  in file:  EG-MSL.png .....

## Ok!
## 
## Performing AMOVA
## AMOVA TABLE  d.f.    SSD         MSD         Variance
## among groups  4   249.4   62.35   2.775
## within groups     42      1528    36.37   36.37
## Total             46      1777    38.63
## 
## Phi_ST =  0.07089   (P<0.0001)
## 
## Pairwise Phi_ST
## ---------------------------------------
## AN  -  CU :  -0.004677     (P= 0.5605 )
## AN  -  FR :  0.00147       (P= 0.4549 )
## AN  -  LM :  0.007328      (P= 0.3287 )
## AN  -  PT :  0.1612    (P= 1e-04 )
## CU  -  FR :  0.01091       (P= 0.2951 )
## CU  -  LM :  0.004841      (P= 0.3682 )
## CU  -  PT :  0.1087    (P<0.0001)
## FR  -  LM :  0.04014       (P= 0.0787 )
## FR  -  PT :  0.1077    (P= 5e-04 )
## LM  -  PT :  0.1924    (P<0.0001)
## 
## 
## *****************************
## Analysis of NML
## 
## - Saving clustering tree figure for NML in file:  EG-NML-NJ.png .....

## Ok!

## Warning in cmdscale(DM, k = length(inds) - 1, eig = T): only 45 of the
first 46
## eigenvalues are > 0

## - Saving PCoA figure for  NML  in file:  EG-NML.png .....
```

```
## Ok!
##
## Performing AMOVA
## AMOVA TABLE  d.f.    SSD         MSD         Variance
## among groups  4   32.6    8.149   0.113
## within groups     42      297.8   7.091   7.091
## Total             46      330.4   7.183
##
## Phi_ST =  0.01569   (P= 0.0422 )
##
## Pairwise Phi_ST
## ------------------------------------
## AN   -  CU :  -0.01757      (P= 0.913 )
## AN   -  FR :  0.008125      (P= 0.2569 )
## AN   -  LM :  0.01819       (P= 0.1257 )
## AN   -  PT :  0.006843      (P= 0.3438 )
## CU   -  FR :  -0.000661     (P= 0.4133 )
## CU   -  LM :  0.02604       (P= 0.0491 )
## CU   -  PT :  0.01469       (P= 0.1361 )
## FR   -  LM :  0.0366    (P= 0.044 )
## FR   -  PT :  0.03928       (P= 0.0077 )
## LM   -  PT :  0.04893       (P= 0.0016 )
##
## Mantel test (MSL/NML): r =  0.4182973  ( P =  0.000999 ; nperm=  1000
)
## Done!
```

AMOVAs comparing regions

```
read_csv(here("raw_data","Genotypes Table_Eggs.csv"), show_col_types =
FALSE) %>%
  mutate("...1"=ifelse(is.element(.$...1,c("FR","PT")), "Northern",
"Southern")) %>%
  write_csv(here("Eggs_region.csv"))

## New names:
## * `` -> ...1
## * `` -> ...2
## * `` -> ...3

res_EG_region <- msap(here("Eggs_region.csv"),
name="EG_region",do.pairwisePhiST = F,do.mantel = F,do.cluster = F)

##
## msap  1.1.9  - Statistical analysis for Methylation-Sensitive
Amplification Polimorphism data
##
## Reading  /Users/andres/Dropbox/tasks/Taliepus/Eggs_region.csv .....
Ok!
## Number of loci:  610
```

```
## Number of samples/individuals:  47
## Number of groups/populations:  2
## Number of primer combinations:  1
## Loci per primer combinations  610
## Error rates per primer combination:  0.05
## Primer:  1
## --Number of Methylation-Susceptible Loci (MSL):  418
## --Number of No Methylated Loci (NML):  192
##
## All combinations:
## Number of Methylation-Susceptible Loci (MSL):  418
## Number of No Methylated Loci (NML):  192
##
## Number of polymorphic MSL:  256  ( 61 % of total MSL)
## Number of polymorphic NML:  144  ( 75 % of total NML)
##
## - Saving transformed matrix for MSL in file:  EG_region-MSL-
transformed.csv
## - Saving transformed matrix for NML in file:  EG_region-NML-
transformed.csv
##
## Shannon's Diversity Index
## MSL: I =  0.4035468   (SD:  0.1824195 )
## NML: I =  0.1984176   (SD:  0.07253319 )
## Wilcoxon rank sum test with continuity correction :  W  =  30310  ( P
< 0.0001 )
##
##
## *****************************
## Analysis of MSL
## Report of methylation levels
##                                                   Northern Southern
## HPA+/MSP+ (Unmethylated)                           0.08289   0.0992
## HPA+/MSP- (Hemimethylated)                         0.22558   0.2443
## HPA-/MSP+ (Internal cytosine methylation)          0.16254   0.1884
## HPA-/MSP- (Full methylation or absence of target)  0.52899   0.4681

## Warning in cmdscale(DM, k = length(inds) - 1, eig = T): only 43 of the
first 46
## eigenvalues are > 0

## - Saving PCoA figure for  MSL  in file:  EG_region-MSL.png .....

## Ok!
##
## Performing AMOVA
## AMOVA TABLE  d.f.    SSD         MSD        Variance
## among groups  1   101.4   101.4   2.956
## within groups     45      1676    37.23   37.23
## Total             46      1777    38.63
##
```

```
## Phi_ST =  0.07354   (P<0.0001)
##
##
## *****************************
## Analysis of NML
##
## - Saving PCoA figure for  NML  in file:  EG_region-NML.png .....

## Ok!
##
## Performing AMOVA
## AMOVA TABLE  d.f.    SSD         MSD         Variance
## among groups  1   8.002   8.002   0.03857
## within groups     45      322.4   7.165   7.165
## Total             46      330.4   7.183
##
## Phi_ST =  0.005354   (P= 0.2006 )
## Done!
```

Gonads

```
res_GO <- msap(here("raw_data","Genotypes Table_GO.csv"),
name="GO",do.pairwisePhiST = T,do.mantel = T)

##
## msap  1.1.9  - Statistical analysis for Methylation-Sensitive
Amplification Polimorphism data
##
## Reading  /Users/andres/Dropbox/tasks/Taliepus/raw_data/Genotypes
Table_GO.csv ..... Ok!
## Number of loci:  610
## Number of samples/individuals:  46
## Number of groups/populations:  5
## Number of primer combinations:  1
## Loci per primer combinations  610
## Error rates per primer combination:  0.05
## Primer:  1
## --Number of Methylation-Susceptible Loci (MSL):  409
## --Number of No Methylated Loci (NML):  201
##
## All combinations:
## Number of Methylation-Susceptible Loci (MSL):  409
## Number of No Methylated Loci (NML):  201
##
## Number of polymorphic MSL:  269  ( 66 % of total MSL)
## Number of polymorphic NML:  182  ( 91 % of total NML)
##
## - Saving transformed matrix for MSL in file:  GO-MSL-transformed.csv
## - Saving transformed matrix for NML in file:  GO-NML-transformed.csv
```

```
##
## Shannon's Diversity Index
## MSL: I =  0.4208246   (SD:  0.1666505 )
## NML: I =  0.2060372   (SD:  0.0749239 )
## Wilcoxon rank sum test with continuity correction :  W  =  42384.5  (
P < 0.0001 )
##
##
## ****************************
## Analysis of MSL
## Report of methylation levels
##                                                         AN      CU
FR     LM
## HPA+/MSP+ (Unmethylated)                             0.08264 0.08775
0.0468 0.0599
## HPA+/MSP- (Hemimethylated)                           0.14132 0.16191
0.1645 0.1362
## HPA-/MSP+ (Internal cytosine methylation)            0.23985 0.21733
0.1243 0.2193
## HPA-/MSP- (Full methylation or absence of target) 0.53619 0.53301
0.6643 0.5846
##                                                         PT
## HPA+/MSP+ (Unmethylated)                             0.09046
## HPA+/MSP- (Hemimethylated)                           0.22249
## HPA-/MSP+ (Internal cytosine methylation)            0.16430
## HPA-/MSP- (Full methylation or absence of target) 0.52274
##
##
## - Saving clustering tree figure for MSL in file:  GO-MSL-NJ.png .....

## Ok!

## Warning in cmdscale(DM, k = length(inds) - 1, eig = T): only 38 of the
first 45
## eigenvalues are > 0

## - Saving PCoA figure for  MSL  in file:  GO-MSL.png .....

## Ok!
##
## Performing AMOVA
## AMOVA TABLE  d.f.    SSD         MSD         Variance
## among groups  4   240.3   60.09   2.125
## within groups     41      1665    40.61   40.61
## Total             45      1905    42.34
##
## Phi_ST =  0.04973   (P<0.0001)
##
## Pairwise Phi_ST
## ---------------------------------------
## AN  -  CU :  0.08857       (P= 1e-04 )
```

```
## AN  -  FR :  0.1212    (P= 3e-04 )
## AN  -  LM :  0.1368    (P= 1e-04 )
## AN  -  PT :  0.07506       (P= 0.0029 )
## CU  -  FR :  -0.05027      (P= 0.96 )
## CU  -  LM :  0.004911      (P= 0.4001 )
## CU  -  PT :  0.01383       (P= 0.2337 )
## FR  -  LM :  -0.03538      (P= 0.8873 )
## FR  -  PT :  0.02133       (P= 0.2223 )
## LM  -  PT :  0.07048       (P= 0.0053 )
##
##
## *****************************
## Analysis of NML
##
## - Saving clustering tree figure for NML in file:  GO-NML-NJ.png .....

## Ok!

## Warning in cmdscale(DM, k = length(inds) - 1, eig = T): only 44 of the
first 45
## eigenvalues are > 0

## - Saving PCoA figure for  NML  in file:  GO-NML.png .....

## Ok!
##
## Performing AMOVA
## AMOVA TABLE  d.f.    SSD         MSD         Variance
## among groups  4   41.88   10.47   0.1122
## within groups     41      387.1   9.441   9.441
## Total             45      429     9.532
##
## Phi_ST =  0.01174   (P= 0.1137 )
##
## Pairwise Phi_ST
## ------------------------------------
## AN  -  CU :  0.05911       (P= 0.0041 )
## AN  -  FR :  0.05445       (P= 0.0076 )
## AN  -  LM :  -0.006247     (P= 0.7377 )
## AN  -  PT :  -0.0002218    (P= 0.5457 )
## CU  -  FR :  0.003647      (P= 0.3288 )
## CU  -  LM :  -0.006874     (P= 0.5506 )
## CU  -  PT :  0.01868       (P= 0.1546 )
## FR  -  LM :  -0.024    (P= 0.9368 )
## FR  -  PT :  0.03728       (P= 0.0357 )
## LM  -  PT :  -0.001285     (P= 0.5505 )
##
## Mantel test (MSL/NML): r =  0.2286962  ( P =  0.01299 ; nperm=  1000 )
## Done!
```

AMOVAs comparing regions

```
  read_csv(here("raw_data","Genotypes Table_GO.csv"), show_col_types = 
FALSE) %>%
  mutate("...1"=ifelse(is.element(.$...1,c("FR","PT")), "Northern", 
"Southern")) %>%
  write_csv(here("GO_region.csv"))

## New names:
## * `` -> ...1
## * `` -> ...2
## * `` -> ...3

res_EG_region <- msap(here("GO_region.csv"), 
name="GO_region",do.pairwisePhiST = F,do.mantel = F,do.cluster = F)

## 
## msap  1.1.9  - Statistical analysis for Methylation-Sensitive 
Amplification Polimorphism data
## 
## Reading  /Users/andres/Dropbox/tasks/Taliepus/GO_region.csv ..... Ok!
## Number of loci:  610
## Number of samples/individuals:  46
## Number of groups/populations:  2
## Number of primer combinations:  1
## Loci per primer combinations  610
## Error rates per primer combination:  0.05
## Primer:  1
## --Number of Methylation-Susceptible Loci (MSL):  409
## --Number of No Methylated Loci (NML):  201
## 
## All combinations:
## Number of Methylation-Susceptible Loci (MSL):  409
## Number of No Methylated Loci (NML):  201
## 
## Number of polymorphic MSL:  269  ( 66 % of total MSL)
## Number of polymorphic NML:  182  ( 91 % of total NML)
## 
## - Saving transformed matrix for MSL in file:  GO_region-MSL-
transformed.csv
## - Saving transformed matrix for NML in file:  GO_region-NML-
transformed.csv
## 
## Shannon's Diversity Index
## MSL: I =  0.4208246   (SD:  0.1666505 )
## NML: I =  0.2060372   (SD:  0.0749239 )
## Wilcoxon rank sum test with continuity correction :  W  =  42384.5  ( 
P < 0.0001 )
## 
## 
```

```
## ****************************
## Analysis of MSL
## Report of methylation levels
##                                                    Northern  Southern
## HPA+/MSP+ (Unmethylated)                            0.07249   0.07638
## HPA+/MSP- (Hemimethylated)                          0.19862   0.14594
## HPA-/MSP+ (Internal cytosine methylation)           0.14785   0.22578
## HPA-/MSP- (Full methylation or absence of target)   0.58104   0.55189

## Warning in cmdscale(DM, k = length(inds) - 1, eig = T): only 38 of the
first 45
## eigenvalues are > 0

## - Saving PCoA figure for  MSL  in file:  GO_region-MSL.png .....

## Ok!
##
## Performing AMOVA
## AMOVA TABLE  d.f.    SSD          MSD          Variance
## among groups  1   46.25   46.25   0.1862
## within groups     44      1859    42.25   42.25
## Total             45      1905    42.34
##
## Phi_ST =  0.004388   (P= 0.3112 )
##
##
## ****************************
## Analysis of NML

## Warning in cmdscale(DM, k = length(inds) - 1, eig = T): only 43 of the
first 45
## eigenvalues are > 0

## - Saving PCoA figure for  NML  in file:  GO_region-NML.png .....

## Ok!
##
## Performing AMOVA
## AMOVA TABLE  d.f.    SSD          MSD          Variance
## among groups  1   7.821   7.821   -0.08167
## within groups     44      421.1   9.571   9.571
## Total             45      429     9.532
##
## Phi_ST =  -0.008607   (P= 0.9062 )
## Done!
```

Muscle

```
res_MU <- msap(here("raw_data","Genotypes Table_MU.csv"),
name="MU",do.pairwisePhiST = T,do.mantel = T)
```

```
##
## msap  1.1.9  - Statistical analysis for Methylation-Sensitive
Amplification Polimorphism data
##
## Reading  /Users/andres/Dropbox/tasks/Taliepus/raw_data/Genotypes
Table_MU.csv ..... Ok!
## Number of loci:  610
## Number of samples/individuals:  47
## Number of groups/populations:  5
## Number of primer combinations:  1
## Loci per primer combinations  610
## Error rates per primer combination:  0.05
## Primer:  1
## --Number of Methylation-Susceptible Loci (MSL):  406
## --Number of No Methylated Loci (NML):  204
##
## All combinations:
## Number of Methylation-Susceptible Loci (MSL):  406
## Number of No Methylated Loci (NML):  204
##
## Number of polymorphic MSL:  282  ( 69 % of total MSL)
## Number of polymorphic NML:  172  ( 84 % of total NML)
##
## - Saving transformed matrix for MSL in file:  MU-MSL-transformed.csv
## - Saving transformed matrix for NML in file:  MU-NML-transformed.csv
##
## Shannon's Diversity Index
## MSL: I =  0.4079398   (SD:  0.1690709 )
## NML: I =  0.1956234   (SD:  0.07449212 )
## Wilcoxon rank sum test with continuity correction :  W  =  42191  ( P
< 0.0001 )
##
##
## ****************************
## Analysis of MSL
## Report of methylation levels
##                                                      AN      CU
FR
## HPA+/MSP+ (Unmethylated)                         0.08473 0.08812
0.07174
## HPA+/MSP- (Hemimethylated)                       0.15640 0.21045
0.16656
## HPA-/MSP+ (Internal cytosine methylation)        0.26552 0.22332
0.16472
## HPA-/MSP- (Full methylation or absence of target) 0.49335 0.47811
0.59698
##                                                      LM     PT
## HPA+/MSP+ (Unmethylated)                         0.09158 0.1040
## HPA+/MSP- (Hemimethylated)                       0.15338 0.2805
## HPA-/MSP+ (Internal cytosine methylation)        0.16391 0.1765
```

```
## HPA-/MSP- (Full methylation or absence of target) 0.59113 0.4390
##
##
## - Saving clustering tree figure for MSL in file:  MU-MSL-NJ.png .....

## Ok!

## Warning in cmdscale(DM, k = length(inds) - 1, eig = T): only 43 of the
first 46
## eigenvalues are > 0

## - Saving PCoA figure for  MSL  in file:  MU-MSL.png .....

## Ok!
##
## Performing AMOVA
## AMOVA TABLE  d.f.    SSD         MSD         Variance
## among groups  4   234.2   58.56   1.652
## within groups     42      1809    43.08   43.08
## Total             46      2044    44.43
##
## Phi_ST =  0.03693   (P= 8e-04 )
##
## Pairwise Phi_ST
## ------------------------------------
## AN  -  CU :  0.02529       (P= 0.074 )
## AN  -  FR :  0.1092    (P= 4e-04 )
## AN  -  LM :  0.1098    (P<0.0001)
## AN  -  PT :  0.08199       (P= 5e-04 )
## CU  -  FR :  0.01966       (P= 0.2107 )
## CU  -  LM :  0.01038       (P= 0.301 )
## CU  -  PT :  0.0178    (P= 0.1907 )
## FR  -  LM :  -0.01825      (P= 0.7624 )
## FR  -  PT :  0.007865      (P= 0.3511 )
## LM  -  PT :  0.0001176     (P= 0.4536 )
##
##
## *****************************
## Analysis of NML
##
## - Saving clustering tree figure for NML in file:  MU-NML-NJ.png .....

## Ok!
## - Saving PCoA figure for  NML  in file:  MU-NML.png .....

## Ok!
##
## Performing AMOVA
## AMOVA TABLE  d.f.    SSD         MSD         Variance
## among groups  4   42.22   10.55   0.2466
## within groups     42      346.2   8.243   8.243
```

```
## Total              46      388.4   8.444
##
## Phi_ST =  0.02905   (P= 2e-04 )
##
## Pairwise Phi_ST
## ------------------------------------
## AN  -  CU :  0.01272       (P= 0.1372 )
## AN  -  FR :  0.03077       (P= 0.0279 )
## AN  -  LM :  0.008858      (P= 0.2171 )
## AN  -  PT :  0.02177       (P= 0.1064 )
## CU  -  FR :  0.02379       (P= 0.0509 )
## CU  -  LM :  0.03313       (P= 0.0098 )
## CU  -  PT :  0.03867       (P= 0.0117 )
## FR  -  LM :  0.03604       (P= 0.0403 )
## FR  -  PT :  0.02052       (P= 0.1229 )
## LM  -  PT :  0.0548    (P= 0.0031 )
##
## Mantel test (MSL/NML): r =  0.2286327  ( P =  0.04396 ; nperm=  1000 )
## Done!
```

AMOVAs comparing regions

```
read_csv(here("raw_data","Genotypes Table_MU.csv"), show_col_types =
FALSE) %>%
  mutate("...1"=ifelse(is.element(.$...1,c("FR","PT")), "Northern",
"Southern")) %>%
  write_csv(here("MU_region.csv"))
```

```
## New names:
## * `` -> ...1
## * `` -> ...2
## * `` -> ...3
```

```
res_EG_region <- msap(here("MU_region.csv"),
name="MU_region",do.pairwisePhiST = F,do.mantel = F,do.cluster = F)
```

```
##
## msap  1.1.9  - Statistical analysis for Methylation-Sensitive
Amplification Polimorphism data
##
## Reading  /Users/andres/Dropbox/tasks/Taliepus/MU_region.csv ..... Ok!
## Number of loci:  610
## Number of samples/individuals:  47
## Number of groups/populations:  2
## Number of primer combinations:  1
## Loci per primer combinations  610
## Error rates per primer combination:  0.05
## Primer:  1
```

```
## --Number of Methylation-Susceptible Loci (MSL):  400
## --Number of No Methylated Loci (NML):  210
##
## All combinations:
## Number of Methylation-Susceptible Loci (MSL):  400
## Number of No Methylated Loci (NML):  210
##
## Number of polymorphic MSL:  313  ( 78 % of total MSL)
## Number of polymorphic NML:  178  ( 85 % of total NML)
##
## - Saving transformed matrix for MSL in file:  MU_region-MSL-
transformed.csv
## - Saving transformed matrix for NML in file:  MU_region-NML-
transformed.csv
##
## Shannon's Diversity Index
## MSL: I =  0.4504965   (SD:  0.1394956 )
## NML: I =  0.200245   (SD:  0.07835647 )
## Wilcoxon rank sum test with continuity correction :  W  =  52637.5  (
P < 0.0001 )
##
##
## ****************************
## Analysis of MSL
## Report of methylation levels
##                                                    Northern Southern
## HPA+/MSP+ (Unmethylated)                              0.1028   0.1036
## HPA+/MSP- (Hemimethylated)                            0.2154   0.1593
## HPA-/MSP+ (Internal cytosine methylation)             0.1540   0.2072
## HPA-/MSP- (Full methylation or absence of target)    0.5278   0.5299

## Warning in cmdscale(DM, k = length(inds) - 1, eig = T): only 42 of the
first 46
## eigenvalues are > 0

## - Saving PCoA figure for  MSL  in file:  MU_region-MSL.png .....

## Ok!
##
## Performing AMOVA
## AMOVA TABLE  d.f.    SSD         MSD         Variance
## among groups  1   52.24   52.24   -0.1757
## within groups     45      2523    56.06   56.06
## Total             46      2575    55.97
##
## Phi_ST =  -0.003143   (P= 0.5479 )
##
##
## ****************************
## Analysis of NML
```

```
##
## - Saving PCoA figure for  NML  in file:  MU_region-NML.png .....

## Ok!
##
## Performing AMOVA
## AMOVA TABLE  d.f.    SSD         MSD         Variance
## among groups  1   15.7    15.7    0.3148
## within groups     45      399.1   8.87    8.87
## Total             46      414.9   9.019
##
## Phi_ST =  0.03428   (P= 9e-04 )
## Done!
```

Gills

```
#msap analysis for Eggs
res_GI <- msap(here("raw_data","Genotypes Table_GI.csv"),
name="GI",do.pairwisePhiST = T,do.mantel = T)

##
## msap  1.1.9  - Statistical analysis for Methylation-Sensitive
Amplification Polimorphism data
##
## Reading  /Users/andres/Dropbox/tasks/Taliepus/raw_data/Genotypes
Table_GI.csv ..... Ok!
## Number of loci:  610
## Number of samples/individuals:  48
## Number of groups/populations:  5
## Number of primer combinations:  1
## Loci per primer combinations  610
## Error rates per primer combination:  0.05
## Primer:  1
## --Number of Methylation-Susceptible Loci (MSL):  481
## --Number of No Methylated Loci (NML):  129
##
## All combinations:
## Number of Methylation-Susceptible Loci (MSL):  481
## Number of No Methylated Loci (NML):  129
##
## Number of polymorphic MSL:  329  ( 68 % of total MSL)
## Number of polymorphic NML:  124  ( 96 % of total NML)
##
## - Saving transformed matrix for MSL in file:  GI-MSL-transformed.csv
## - Saving transformed matrix for NML in file:  GI-NML-transformed.csv
##
## Shannon's Diversity Index
## MSL: I =  0.4481987   (SD:  0.1510266 )
## NML: I =  0.1995668   (SD:  0.06758407 )
```

```
## Wilcoxon rank sum test with continuity correction :  W  =  37740.5  (
P < 0.0001 )
##
##
## ****************************
## Analysis of MSL
## Report of methylation levels
##                                                        AN      CU
FR
## HPA+/MSP+ (Unmethylated)                            0.0817 0.07838
0.06471
## HPA+/MSP- (Hemimethylated)                          0.1622 0.15322
0.13981
## HPA-/MSP+ (Internal cytosine methylation)           0.1911 0.20811
0.16892
## HPA-/MSP- (Full methylation or absence of target) 0.5651 0.56029
0.62656
##                                                         LM      PT
## HPA+/MSP+ (Unmethylated)                            0.07651 0.09044
## HPA+/MSP- (Hemimethylated)                          0.17921 0.19168
## HPA-/MSP+ (Internal cytosine methylation)           0.14449 0.18358
## HPA-/MSP- (Full methylation or absence of target) 0.59979 0.53430
##
##
## - Saving clustering tree figure for MSL in file:  GI-MSL-NJ.png .....

## Ok!

## Warning in cmdscale(DM, k = length(inds) - 1, eig = T): only 43 of the
first 47
## eigenvalues are > 0

## - Saving PCoA figure for  MSL  in file:  GI-MSL.png .....

## Ok!
##
## Performing AMOVA
## AMOVA TABLE  d.f.    SSD         MSD         Variance
## among groups  4   275.7   68.94   1.582
## within groups     43      2312    53.78   53.78
## Total             47      2588    55.07
##
## Phi_ST =  0.02857   (P= 0.002 )
##
## Pairwise Phi_ST
## ---------------------------------------
## AN  -  CU :  0.06195       (P= 0.0043 )
## AN  -  FR :  0.06664       (P= 0.0087 )
## AN  -  LM :  0.01106       (P= 0.2489 )
## AN  -  PT :  0.04851       (P= 0.007 )
## CU  -  FR :  0.01028       (P= 0.3148 )
```

```
## CU  -  LM :  0.02187       (P= 0.1141 )
## CU  -  PT :  0.0276    (P= 0.0614 )
## FR  -  LM :  -0.007319     (P= 0.6215 )
## FR  -  PT :  0.04733       (P= 0.0133 )
## LM  -  PT :  -0.0001676    (P= 0.5026 )
##
##
## *****************************
## Analysis of NML
##
## - Saving clustering tree figure for NML in file:  GI-NML-NJ.png .....

## Ok!

## Warning in cmdscale(DM, k = length(inds) - 1, eig = T): only 46 of the
first 47
## eigenvalues are > 0

## - Saving PCoA figure for  NML  in file:  GI-NML.png .....

## Ok!
##
## Performing AMOVA
## AMOVA TABLE  d.f.    SSD         MSD         Variance
## among groups  4   29.12   7.28    0.1226
## within groups     43      262.5   6.105   6.105
## Total             47      291.6   6.205
##
## Phi_ST =  0.01969   (P= 0.028 )
##
## Pairwise Phi_ST
## -----------------------------------
## AN  -  CU :  0.01382       (P= 0.1787 )
## AN  -  FR :  -0.02041      (P= 0.9342 )
## AN  -  LM :  0.0252    (P= 0.1053 )
## AN  -  PT :  -0.01689      (P= 0.9415 )
## CU  -  FR :  0.01798       (P= 0.1324 )
## CU  -  LM :  0.0488    (P= 0.0425 )
## CU  -  PT :  0.009421      (P= 0.2865 )
## FR  -  LM :  0.01208       (P= 0.2013 )
## FR  -  PT :  -0.002803     (P= 0.5742 )
## LM  -  PT :  0.0426    (P= 0.0451 )
##
## Mantel test (MSL/NML): r =  0.04227242  ( P =  0.3137 ; nperm=  1000 )
## Done!
```

AMOVAs comparing regions

```
read_csv(here("raw_data","Genotypes Table_GI.csv"), show_col_types =
FALSE) %>%
  mutate("...1"=ifelse(is.element(.$...1,c("FR","PT")), "Northern",
"Southern")) %>%
  write_csv(here("GI_region.csv"))

## New names:
## * `` -> ...1
## * `` -> ...2
## * `` -> ...3

res_EG_region <- msap(here("GI_region.csv"),
name="GI_region",do.pairwisePhiST = F,do.mantel = F,do.cluster = F)

##
## msap  1.1.9  - Statistical analysis for Methylation-Sensitive
Amplification Polimorphism data
##
## Reading  /Users/andres/Dropbox/tasks/Taliepus/GI_region.csv ..... Ok!
## Number of loci:  610
## Number of samples/individuals:  48
## Number of groups/populations:  2
## Number of primer combinations:  1
## Loci per primer combinations  610
## Error rates per primer combination:  0.05
## Primer:  1
## --Number of Methylation-Susceptible Loci (MSL):  481
## --Number of No Methylated Loci (NML):  129
##
## All combinations:
## Number of Methylation-Susceptible Loci (MSL):  481
## Number of No Methylated Loci (NML):  129
##
## Number of polymorphic MSL:  329  ( 68 % of total MSL)
## Number of polymorphic NML:  124  ( 96 % of total NML)
##
## - Saving transformed matrix for MSL in file:  GI_region-MSL-
transformed.csv
## - Saving transformed matrix for NML in file:  GI_region-NML-
transformed.csv
##
## Shannon's Diversity Index
## MSL: I =  0.4481987   (SD:  0.1510266 )
## NML: I =  0.1995668   (SD:  0.06758407 )
## Wilcoxon rank sum test with continuity correction :  W  =  37740.5  (
P < 0.0001 )
```

```
##
##
## ****************************
## Analysis of MSL
## Report of methylation levels
##                                                   Northern Southern
## HPA+/MSP+ (Unmethylated)                            0.0790  0.07886
## HPA+/MSP- (Hemimethylated)                          0.1686  0.16486
## HPA-/MSP+ (Internal cytosine methylation)           0.1771  0.18122
## HPA-/MSP- (Full methylation or absence of target)   0.5753  0.57505

## Warning in cmdscale(DM, k = length(inds) - 1, eig = T): only 42 of the
first 47
## eigenvalues are > 0

## - Saving PCoA figure for  MSL  in file:  GI_region-MSL.png .....

## Ok!
##
## Performing AMOVA
## AMOVA TABLE  d.f.    SSD          MSD         Variance
## among groups  1   56.01   56.01   0.04282
## within groups     46      2532    55.05   55.05
## Total             47      2588    55.07
##
## Phi_ST =  0.0007772   (P= 0.4361 )
##
##
## ****************************
## Analysis of NML

## Warning in cmdscale(DM, k = length(inds) - 1, eig = T): only 43 of the
first 47
## eigenvalues are > 0

## - Saving PCoA figure for  NML  in file:  GI_region-NML.png .....

## Ok!
##
## Performing AMOVA
## AMOVA TABLE  d.f.    SSD          MSD         Variance
## among groups  1   5.835   5.835   -0.01683
## within groups     46      285.8   6.213   6.213
## Total             47      291.6   6.205
##
## Phi_ST =  -0.002715   (P= 0.5773 )
## Done!
```

Single locus analyses and heatmaps

Here, locus-per-locus chi-squared tests are performed as an approximation to check which loci the different methylation patterns (h,i,u) are not randomly distributed between the two regions (Northern and Southern). We select only those loci with $p<0.05$ and plotted them in a heatmap ordering them by increasing p

```
# Function to make a heatmap with the methylation patterns from a
res_msap object
# It uses those loci that showed significant differences in the Chi
square test between regions (Northern and Southern)
plot_HeatMap <- function(MSAP, threshold=0.05, cluster_rows = F,
cluster_columns = F){

  # Ordenate the sites (groups) by latitude and define both regions
  localities <- factor(MSAP$groups, levels=c("FR","PT","CU","LM","AN"))
  regions <- ifelse(is.element(localities,c("FR","PT")), "Northern",
"Southern")

  # get the methylation patterns
  methpatterns <- do.call(rbind.data.frame, MSAP$patterns)
  methpatterns[methpatterns=="f"] <- NA
  methpatterns <- as.data.frame(unclass(methpatterns))
  filter2 <-apply(methpatterns,2,
FUN=function(X){ifelse(length(levels(factor(X)))>=2,T,F)})
  #Apply the fisher test to all the loci
  pvals2<- apply(methpatterns[,filter2],2,
FUN=function(X){suppressWarnings(chisq.test(
                  table(regions,X))$p.value)})

  selected <- na.omit(names(pvals2[pvals2<threshold]))

  ordered_patterns <- methpatterns[order(localities),selected]
  ordered_patterns <- ordered_patterns[ ,order(pvals2[selected])]


  #Plotting
  anno_df <- data.frame( Site=localities, Region=regions)
  anno_df <- anno_df[order(localities),]
  color_anno = list( Region = c("Northern"="mediumvioletred",
"Southern"="darkolivegreen"), #Change as needed
                     Site = c( "FR"= "magenta" ,"PT"="mediumorchid1",
                               "CU"="seagreen1", "LM"="yellowgreen",
"AN" = "forestgreen") )

  ha1 <- rowAnnotation(df = anno_df, col = color_anno)
  ha2 <- HeatmapAnnotation('p values'=
anno_barplot(sort(pvals2[selected]),border = F))
  ht1 <- Heatmap(as.matrix(ordered_patterns),name= "Methylation pattern",
```

```
cluster_rows = cluster_rows, cluster_columns = cluster_columns,
                  right_annotation = ha1, row_split = anno_df, row_title =
NULL, show_row_names = F, row_gap = unit(0.8,"mm"),
                  top_annotation = ha2, show_column_names = F,
                  column_title = "MSAP loci", column_title_side =
"bottom",
                  col = c("i"="cornflowerblue", "h"="orange",
"u"="indianred1"), na_col = "gray90")
  return(ht1)
}
```

EGG

```
 plot_HeatMap(res_EG)
```

GILL

```
 plot_HeatMap(res_GI)
```

GONAD

```
 plot_HeatMap(res_GO)
```

MUSCLE

```
   plot_HeatMap(res_MU)
```
