# Supplementary material for: Long term environmental variability modulates the epigenetics of maternal traits of kelp crabs in the coast of Chile
Source: Sci Rep. 2022 Nov 5;12:18806. doi: 10.1038/s41598-022-23165-1 (PMC9637151; doi:10.1038/s41598-022-23165-1)
Supplement: Supplementary file 4 — Supplementary Table S1. [file 41598_2022_23165_MOESM4_ESM.pdf]

Long term environmental variability modulates the epigenetics of maternal traits of kelp crabs in the coast of Chile.

Simone Baldanzi, Gonzalo S. Saldías, Cristian A. Vargas, Francesca Porri

Supplementary Table S1

Shannon Diversity Index of Methylation Sensitive Loci (MSL) and Non-Methylated Loci (NML) for each adult tissue and egg. A Wilcoxon Rank sum test is reported along with the *p*-value.

	SI MSL	SI NML	Wilcoxon Test	*p*-value
Egg	0.40±0.18	0.20±0.07	30310	<0.001
Muscle	0.41±0.17	0.20±0.08	42191	<0.0001
Gonad	0.42±0.17	0.20±0.08	42385	<0.0001
Gill	0.45±0.15	0.20±0.07	37741	<0.0001
